# Hfq balances energetic efficiency and antibiotic persistence in *Acinetobacter baumannii*

**DOI:** 10.1128/msystems.00320-25

**Published:** 2025-08-11

**Authors:** Abhiroop Sett, Arsalan Hussain, Srestha Tomar, Ashish Kumar Ray, Ranjana Pathania

**Affiliations:** 1 Department of Biosciences and Bioengineering, Indian Institute of Technology Roorkee 729296, Roorkee, Uttarakhand, India; 2 Centre of Excellence in Disaster Mitigation and Management, Indian Institute of Technology Roorkee 30112, Roorkee, Uttarakhand, India; National Cancer Institute, Bethesda, Maryland, USA

**Keywords:** RNA chaperone Hfq, post-transcriptional regulation, transcriptome, *Acinetobacter baumannii*, *in vivo *antibiotic persistence

## Abstract

The RNA chaperone Hfq of *Acinetobacter baumannii* has been documented in altering antibiotic susceptibility and other optimal stress tolerance capabilities of this pathogen. However, the understanding of whether and how Hfq impacts antibiotic persistence remains unexplored in *A. baumannii*. Consequently, we show that energetic burden on Δ*hfq* cells is imposed due to perturbation of the global transcription of genes, including ones involved in metabolism, secretion systems, and electron transport. As a result, actively growing Δ*hfq* cells demonstrate better survival in the presence of antibiotics with an increased spontaneous persistence phenotype compared with wild-type (WT) cells in the presence of cefepime. Introducing a chromosomal Hfq variant with a mutation in its proximal RNA-binding face (Hfq_F39A_) resulted in an intermediate effect on growth fitness and cefepime persister frequency compared with WT and Δ*hfq*. Additionally, the Hfq_F39A_ persisters exhibit a disrupted proton motive force along with lower metabolic activity and reduced intracellular ATP. Importantly, both Δ*hfq* and Hfq_F39A_ strains were less virulent than WT; however, they exhibited better relative survival in murine lungs compared with the WT when treated with cefepime. However, our findings indicate that although *hfq* deletion increases spontaneous persister frequency against cefepime *in vitro*, its attenuated virulence results in lower organ burden *in vivo*. Hence, highlighting the fact that although targeting Hfq may contribute to increased persistence, its reduced organ burden makes it a clinically relevant drug target.

**IMPORTANCE:**

This study demonstrates the significance of Hfq in modulating physiology and antibiotic persistence of *Acinetobacter baumannii*. By comparing the *hfq* deletion mutant with wild-type *A. baumannii*, we uncovered a persistence mechanism that increases the survival of this pathogen, particularly under cefepime stress, through pleiotropic dysregulation of post-transcriptional networks impacting cellular energetics. In Δ*hfq* strains, the elevated expression of energy-intensive genes (such as those for Type VI and II secretion systems), along with the suppression of metabolic and electron transport pathways, leads to increased persister frequencies. This illustrates how Hfq impacts both growth and antibiotic persistence. Introducing an Hfq variant (Hfq_F39A_) with impaired RNA binding showed an intermediate effect on growth and persistence, further implicating Hfq functionality in antibiotic persistence. Overall, these findings provide a foundation for targeting Hfq in *A. baumannii* therapeutics, with implications for balancing reduced virulence against the risk of increased relative survival to frontline antibiotics, particularly in immunocompromised hosts.

## INTRODUCTION

Research into the treatment of infectious diseases caused by pathogenic bacteria contributed to the dawn of the antibiotic era. Over time, many bacterial pathogens have developed strategies to evade antibiotic treatment. These strategies include resistance, persistence, heteroresistance, and tolerance (1). Although a major focus of research has largely been on antibiotic resistance, recent studies have highlighted the importance of other survival strategies, particularly in recurring infections (1, 2). Among these, antibiotic persistence stands out as a key mechanism by which a bacterial subpopulation survives lethal doses of antibiotics, often leading to treatment failures (2). Bacterial antibiotic persisters, which exhibit non-inheritable tolerance to antibiotics, typically represent a subpopulation of the total bacterial population and are classified into two types: triggered and spontaneous persisters (2, 3). Triggered persisters arise due to external stress, like antibiotic exposure or nutrient limitation, adopting a growth-arrested state, which is lost in the absence of stress or in exponential growth (2). In contrast, spontaneous persisters emerge stochastically, primarily as slowly growing cells, and are continuously generated during the growth phase (2).

Antimicrobial resistance (AMR) leads to higher mortality and significant economic losses (4, 5). Although novel antibiotics and treatments are being developed to combat AMR, treatment failures due to antibiotic persistence present a major challenge, as they may lead to AMR (6, 7). Understanding new mechanisms of bacterial persistence is a promising route toward developing better treatment options in the future. Global regulators of bacterial physiology, such as ProQ and Hfq, the latter of which has been touted as a potential drug target (8), are known to induce persistence in various pathogens (7, 9–12). Recent research in *Salmonella* highlights the critical role of post-transcriptional regulation in antibiotic persistence (12). Specifically, the ProQ protein of *Salmonella sp*. increases persistence by upregulating energetically demanding genes, resulting in a higher frequency of persisters against antibiotics like ampicillin and ciprofloxacin when compared with *proQ* deletion mutants (12). However, in *Escherichia coli*, deletion of *hfq* has been associated with increased antibiotic persistence (9). In contrast, in *Yersinia ruckeri* and *Aeromonas veronii*, *hfq* deletion leads to a loss of antibiotic persistence phenotype (10, 11). The role of post-transcriptional regulation in antibiotic persistence is further illustrated in pathogenic *Staphylococcus aureus*. This bacterium forms small colony variants (SCVs) that persist under aminoglycoside stress and exhibit a differential expression of about 18 sRNAs (13). Hence, studying the role of these RNA chaperones in antibiotic persistence is essential to prevent future treatment failures.

*Acinetobacter baumannii* is a World Health Organization-designated critical pathogen known for causing multi-drug-resistant (MDR) infections (13, 14). This pathogen thrives under diverse environmental stresses both within and outside its host, aiding its establishment as a nosocomial, MDR pathogen (13, 15). This adaptability under varied stress conditions is partly due to post-transcriptional gene regulation (16, 17), a key regulatory mechanism also observed in other gram-negative pathogens such as *E. coli* and *Pseudomonas sp*. (18). Central to this regulation is the RNA chaperone protein Hfq, which accelerates interactions between small RNAs (sRNAs) and their target mRNAs 1,000 times faster than unchaperoned interactions (19, 20). Hfq brings about this interaction by binding to RNA targets via several crucial amino acids on its proximal, distal, and rim faces (21, 22). Although additional RNA-chaperoning proteins like ProQ and CsrA contribute to gene regulation across various bacterial strains (23, 24), Hfq remains the primarily studied RNA chaperone in *A. baumannii*, significantly impacting its pathophysiology (16, 17, 25). Research shows that modifications and deletion of Hfq in *A. baumannii* and other pathogens lead to notable phenotypic changes, such as impaired growth, reduced virulence, lower desiccation tolerance, and increased sensitivity to antibiotics (16, 18, 26). Additionally, in one of our recent studies, we found that deletion of *hfq* imparts an energetic burden via the disruption of membrane integrity, leading to altered proton motive force (PMF) and intracellular ATP levels in *A. baumannii* (25). Although the role of Hfq in virulence and antibiotic sensitivity is documented (16, 26), its influence on antibiotic persistence in *A. baumannii* remains to be fully explored.

Given the slower growth, metabolic defect, and lower intracellular energy pool induced by a *hfq* deletion in *A. baumannii* (25), we investigated its influence on antibiotic persistence. It was already established that deletion of *hfq* causes growth perturbations and lowered cellular ATP levels (25). Hence, we evaluated the ability of a chromosomally complemented *hfq*(c-*hfq*) strain to reverse the growth defect in *A. baumannii hfq* deletion mutant (Δ*hfq*) to wild-type (WT) levels and how *hfq* deletion affects cellular metabolic status in this pathogen. We found that growth was restored in *A. baumannii* c-*hfq* strain, and both the WT and c-*hfq* strains exhibited an optimal metabolic state compared with the Δ*hfq*. Differential transcriptome analysis between WT and *Δhfq* revealed that there is an overall perturbation of transcription in Δ*hfq*, which contributes toward increased ATP demands, lower intracellular NADH, and reduced metabolic flux. These energetic constraints and growth limitations increased the ability of Δ*hfq* to better persist in the presence of different antibiotics compared with WT, especially against the clinically used first-line antibiotic cefepime *in vitro* (27). As Hfq has been targeted as a drug target, we wanted to further confirm the role of Hfq in our observed increased antibiotic persistence. Hence, we introduced an Hfq protein with mutations in its 3’- poly U-rich small RNA-binding site, Hfq_F39A_, into the chromosome of *A. baumannii*. The Hfq_F39A_ face mutant exhibited intermediate persister frequencies compared with WT and Δ*hfq* strains. Notably, loss or modification of an RNA-binding site on Hfq also reduces the virulence of *A. baumannii* in *C. elegans* and murine infection models. However, *hfq* mutants (Hfq_F39A_ and Δ*hfq*) exhibited better survival in murine lungs compared with WT *A. baumannii* when treated with cefepime. However, the overall CFU counts post-cefepime treatment of the *hfq* deletion mutant *in vivo* remained significantly lower than those of WT *A. baumannii*. In conclusion, we have demonstrated that deletion of *hfq* leads to a relative increase in antibiotic survival *in vivo*, particularly against cefepime, with a concomitant loss in virulence in *A. baumannii* inside its host.

## RESULTS

### Deletion of *hfq* impairs growth and alters metabolism

**Fig 1:**
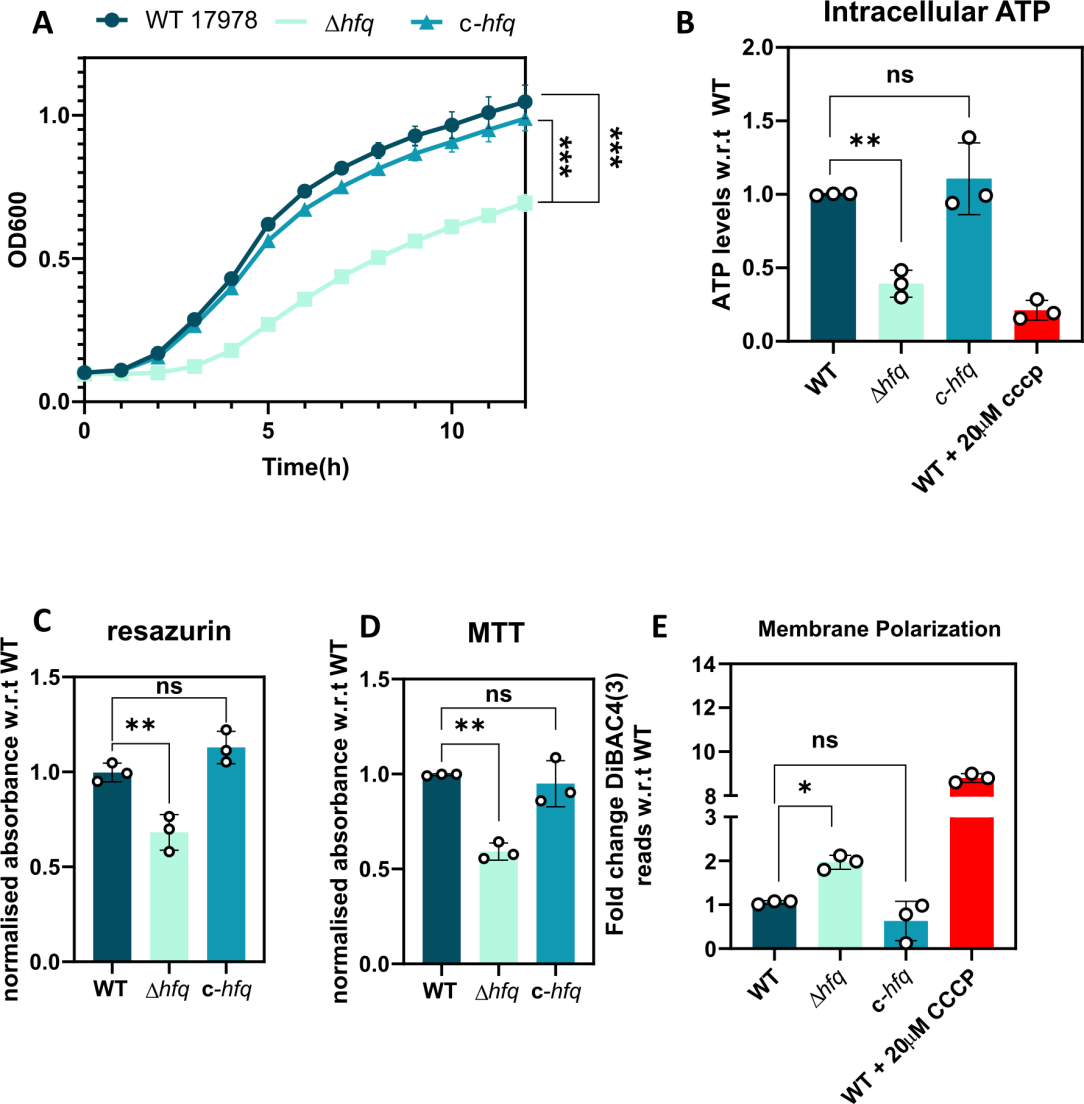
Deletion of *hfq* is accompanied by a growth defect and altered metabolic status of *A. baumannii*. (**A**) A growth profile of all the *A. baumannii* strains grown in LB broth at 37°C at 1 h intervals. Each point represents the mean of four values with SD shown as error bars. The growth phenotype assay is a representative plot of three independent biological replicates. (**B**) Reduction in intracellular ATP levels in the *A. baumannii* WT and Hfq variants grown in LB broth using a luciferase/luciferin assay. Each bar represents the mean of three experiments, and the error bars represent the SD. The CCCP control was not included in the statistical analysis. (**C** and **D**) Accumulation of reducing equivalents like NADH was measured using redox-sensitive resazurin and MTT dyes, respectively. (**E**) Quantification of the degree of membrane depolarization. Measurement of the membrane potential component Δψ was performed using the membrane potential–sensitive fluorescent probe DiBAC4. The CCCP control was not included in the statistical analysis. Each bar represents the mean of three experiments, and the error bars represent the SD. Statistical significance was determined by one-way ANOVA. *, *P* ≤ 0.05; **, *P* ≤ 0.001; ***, *P* ≤ 0.001; ns, non-significant. Tukey’s test was used as a *post hoc* test to determine the statistical significance of all pairs of data. Tukey’s test was used as a *post hoc* test to determine the statistical significance of all pairs of data.

The implication of loss of Hfq-mediated regulation is well documented across different genera of bacterial pathogens (18). A compromised growth phenotype is a hallmark of *hfq* deletion, which is characterized by a longer lag phase followed by a reduced endpoint growth compared with the WT (28). This has also been previously observed by us in the case of *A. baumannii,* where deletion of Hfq induces a compromised growth in enriched media like lysogeny broth (LB), which is further accentuated in single nutrient sources (16). Hence, to conclusively establish the role of Hfq in this phenotype, we evaluated the growth profile of Δ*hfq* and chromosomally complemented *hfq* (c*-hfq*) compared with the WT. We found that chromosomal complementation of Hfq (Fig. S1A and B) restores growth in Δ*hfq* strains (Fig. 1A; Fig. S1C), further supporting our previous observation. This reiterates that the pleiotropic role of *A. baumannii* Hfq is critical for its optimal growth in LB. From earlier reports in *Pseudomonas putida* and our previous findings in *A. baumannii,* growth disruption is accompanied by a lowered ATP level (25, 29). This led us to check whether chromosomal complementation of *hfq* restores the cellular ATP reserve. We found that the intracellular ATP levels of WT and *c-hfq* strains were comparable and maintained at a higher level than the Δ*hfq* strain (Fig. 1B). A contributing factor toward reduced ATP levels is a disrupted metabolic activity of the cell that lowers intracellular concentration of reducing equivalents like NADH (30, 31). Therefore, to test the intracellular NADH levels and overall reducing activity of these strains, we subjected them to resazurin and MTT assays (30, 32–34). We found that the reducing equivalents like NADH in Δ*hfq* are lowered along with a net reduction in ATP levels (Fig. 1C and D), which is in agreement with earlier reports in *P. putida* (29). Reducing equivalents like NADH help in maintaining the cellular proton motive force (PMF), which in turn results in ATP synthesis (35). Therefore, we estimated the membrane polarization of these three strains using a PMF-sensitive dye, DiBAC4. We found that the Δhfq cells exhibited a highly depolarized membrane, that is, a disrupted PMF (Fig. 1E). Hence, the slow growth of the Δ*hfq* cells can be attributed to a reduced metabolic flux that leads to a disrupted PMF, leading to reduced cellular energy levels.

### Deletion of *hfq* induces transcriptome rewiring in *A. baumannii*

**Fig 2:**
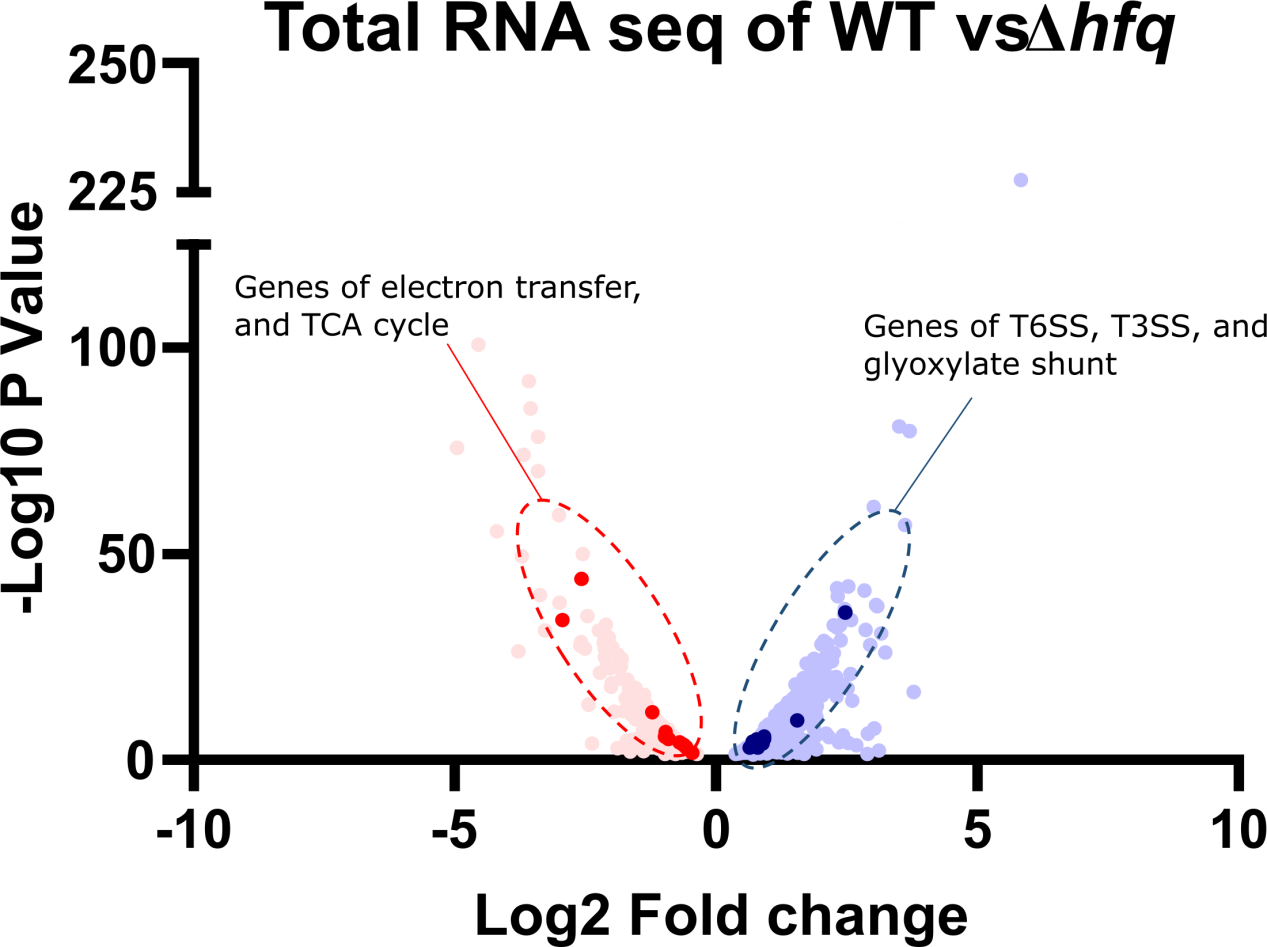
Deletion of *hfq* results in altered transcriptome. A volcano plot depicting statistically significant downregulated (pink) and upregulated (blue) transcript levels of different mRNAs. The genes involved in the TCA cycle and electron transfer are depicted in dark pink, whereas the genes involved in encoding type VI secretion system (T6SS), type III secretion system (T3SS), and the glyoxylate shunt are depicted in dark blue. All data points shown in this figure are statistically significant with a minimum *P*-value of <0.05.

The pleiotropic effect of Hfq on cellular fitness results in growth defects upon its deletion in different gram-negative pathogens, including *A. baumannii* (18, 25). This led us to perform a differential gene expression analysis between mid-log phase cells of WT and Δ*hfq* cells of *A. baumannii*. Our results highlight a significant number of transcripts that are differentially expressed in the Δ*hfq* strain (Fig. S2), listed in Data S1, including that of genes involved in encoding multicomponent secretion systems, electron transport, TCA cycle, and glyoxylate pathway (Fig. 2). The statistically significant upregulated transcripts included that of secretion systems like the type six secretion system (T6SS) (*tssF, tssC, tssG, tssB, tssE,* and *hcp*)*,* type two secretion system (T2SS) (*gspI, gspJ, gspK,* fimbrial protein) (Fig. S3). In our recent study, it was observed that Hfq promotes RNase E-mediated degradation of *tssM* mRNA. The *tssM* gene encodes a T6SS structural component, which is downregulated via interaction with the sRNA AbsR28 with the help of Hfq (36). Therefore, the observed upregulation of *tssM* transcripts in Δ*hfq* may result from disrupted Hfq-AbsR28-*tssM* interactions due to the absence of *hfq* (36). Importantly, the expression of multi-component secretion systems is known to incur an energetic burden on the cell (37). Research has shown that extracellular appendages like secretion system proteins have undergone evolutionary modifications in their amino acid composition so as to reduce the overall energy burden on the cell (37). Hence, overexpression of multicomponent secretion system genes, which are not recycled like the cytosolic proteins, is energetically demanding (37). Moreover, translation genes of the multicomponent type 6 secretion system (T6SS) are known to induce an increased energetic cost (38). In *Salmonella *sp., it was reported that the RNA chaperone ProQ, in its presence, upregulates genes of the flagellar pathway and the type III protein secretion systems. Thus, ProQ confers an increased antibiotic persistence owing to lowered cellular energy levels (12), indicating the probable contribution of increased multicomponent system expression toward our observation.

In contrast, the significantly downregulated genes included those involved in pumping out protons, that is, establishing the proton motive force (PMF). The downregulated transcripts belong to *nuo*-operon encoding respiratory complex I (*nuoC, nuoG, nuoE, nuoF*)*,* cytochrome ubiquinol oxidase subunit I, cytochrome o ubiquinol oxidase subunit IV, cytochrome o ubiquinol oxidase subunit III, and ubiquinol oxidase subunit II (Fig. S3). Hence, the global transcriptome points at the underlying contributors of disrupted PMF and lowered intracellular ATP and NADH levels in *A. baumannii* Δ*hfq* cells. In addition, the results from our previous study also point to a perturbed metabolic state of the Δ*hfq* cells (16, 25). Therefore, we looked at the differential transcript levels of genes involved in metabolism. We found that transcripts of the TCA cycle genes are significantly downregulated (*icd*, *gltA, sucD, sucC, ace, acnA,* and *fumC*), whereas genes of the glyoxylate shunt were upregulated (isocitrate lyase and malate synthase) (Fig. S3). This result suggests that Δ*hfq* strain may channel its metabolic intermediates through the glyoxylate pathway, in turn by passing the TCA cycle and generating two fewer NADH molecules (Fig. S4) (39). Hence, deletion of Hfq perturbs the metabolic state of *A. baumannii,* along with increasing energetic burden on the cell with a concomitant decrease in ATP production.

### Deletion of *hfq* induces increased persistence frequency against antibiotics like cefepime

**Fig 3:**
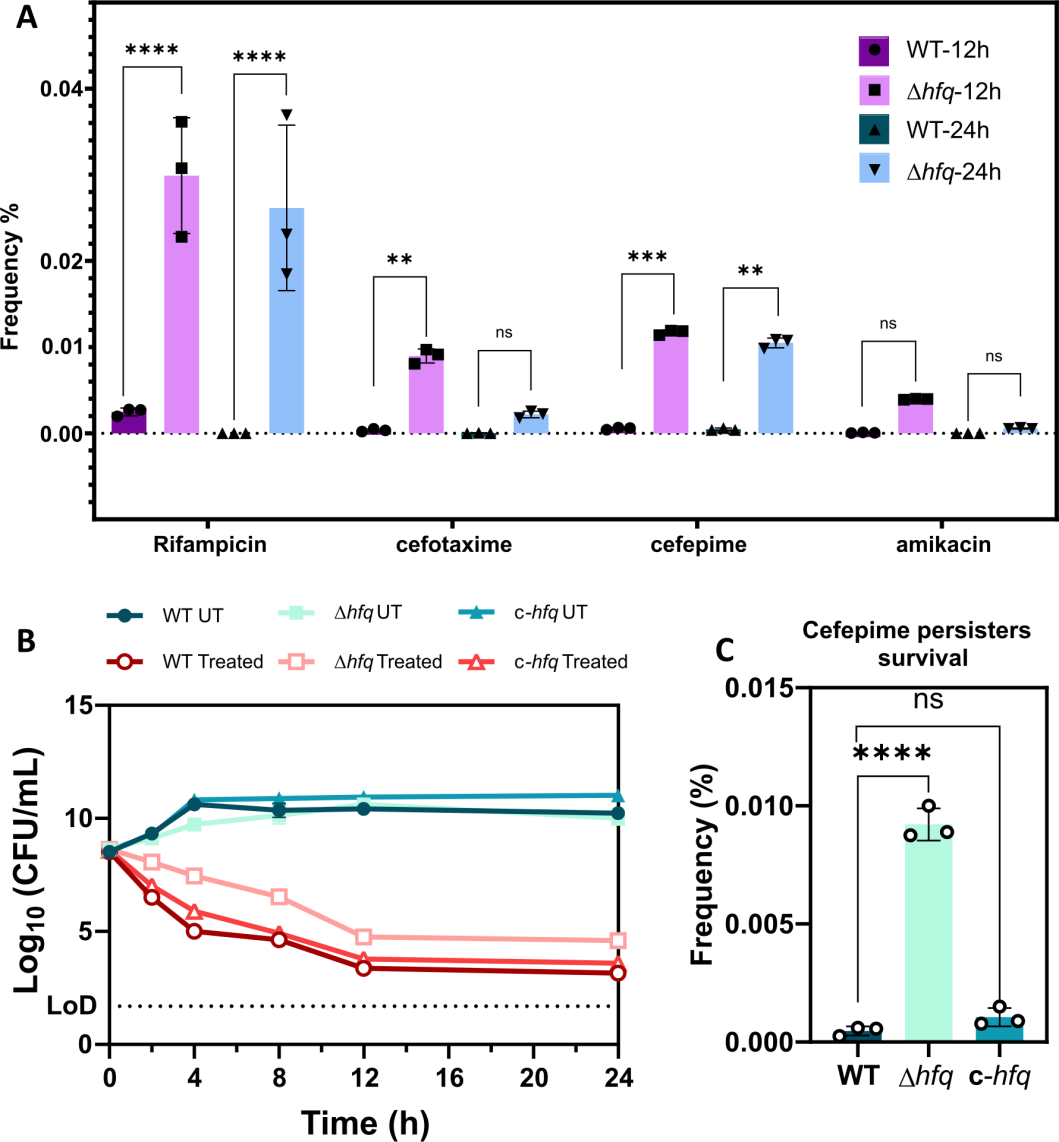
Loss of *hfq* leads to increased spontaneous persistence of *A. baumannii* against different antibiotics. (**A**) The survival percentage of *A. baumannii* wild-type and *hfq* deletion strains post 12 h and 24 h treatment with 50× MIC concentrations of rifampicin (100 mg/L); cefotaxime (400 mg/L); cefepime (400 mg/L); and amikacin (1,600 mg/L). Each bar represents the mean values of three independent biological replicates. Statistical significance was determined by two-way ANOVA. **, *P* ≤ 0.001; ***, *P* ≤ 0.001; ****, *P* ≤ 0.0001; ns, non-significant. Tukey’s test was used as a *post hoc* test to determine the statistical significance of all pairs of data. (**B**) Bi-phasic kill curve of *A. baumannii* wild-type and Hfq mutants (Δ*hfq*) and complemented strain (*c-hfq*) treated with cefepime at 50X MIC (400 mg/L). The bi-phasic kill curve is one representative plot of three independent biological replicates. (**C**) Frequency % of persister formation in response to 50× MIC of cefepime (400 mg/L) calculated as (colonies formed in persister at 24 h/no. of colonies of untreated log phase cells at 0 h) × 100. Each bar represents the mean values of three independent biological replicates. Statistical significance was determined by one-way ANOVA. ****, *P* ≤ 0.0001; ns, non-significant. Tukey’s test was used as a *post hoc* test to determine the statistical significance of all pairs of data.

Compromised growth, lowered intracellular ATP levels, and perturbed metabolic status of a cell are typically involved in conferring bacterial cells with the ability to persist under antibiotic stress (1, 7). Given the perturbed growth phenotype and lowered metabolic activity of log-phase Δ*hfq* cells, we hypothesized that this might impact the ability of these cells to persist under antibiotic stress. To garner further insight, we first determined the minimum inhibitory concentration of different antibiotics for WT and Δ*hfq* strains (Table S4). Subsequently, we carried out time-kill kinetics of log-phase WT cells against different classes of antibiotics at 50× and 100× MIC (Fig. S5). As the WT exhibited better survival at 50× MIC, we compared the survival percentage of log-phase cells of WT and Δ*hfq* strains in the presence of 50× concentration of different antibiotics at 12 h and 24 h post-treatment. We found that Δ*hfq* shows a better propensity to persist or tolerate antibiotic stress than the WT cells (Fig. 3A). Both the WT and Δ*hfq* cells exhibited a spontaneous persistence phenotype till 24 h upon treatment with 50× cefepime, whereas in the case of other antibiotics, the WT cells either failed to persist or did not survive post 12 h.

The broad-spectrum cephalosporin, cefepime, is used as a first-line antibiotic to treat susceptible *A. baumannii* infections and also during treatment of meningitis caused by this pathogen (27, 40). This factor further led us to determine whether deletion of *hfq* has any effect on the ability of this pathogen to persist under lethal doses of cefepime. We found that Δ*hfq* shows a better propensity to persist or tolerate cefepime stress than the WT cells, exhibiting a typical bi-phasic kill curve (Fig. 3B). The Δ*hfq* strain exhibited an increased spontaneous persister frequency compared with WT at 24 h post-treatment with 50× MIC concentration of cefepime (Fig. 3C). As antibiotic persistence is a non-inheritable phenomenon, we also determined whether these strains of *A. baumannii* progressively exhibit higher persistence frequency after each round of cefepime exposure. We found that the frequency of persistence of these WT and Δ*hfq* strains did not show any statistically significant change even after multiple rounds of treatment (Fig. S6). This finding indicates that repeated exposure to cefepime does not lead to inheritable genetic mutations or enhanced persister frequency in these cells.

**Fig 4:**
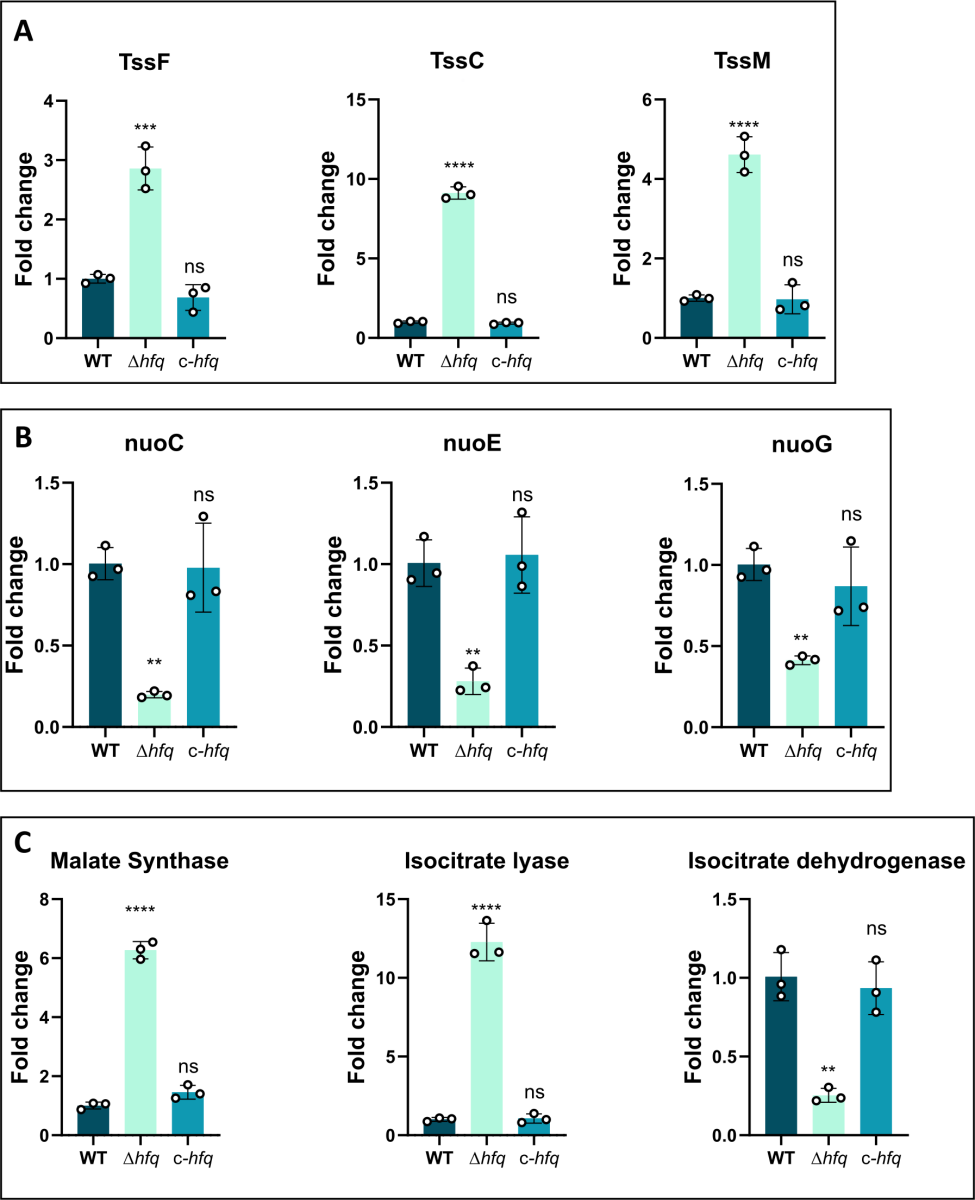
A multitude of genes are differentially expressed in cefepime-induced persisters of *A. baumannii* after 24 h post-treatment. (**A**) The expression profiles of several Type VI secretion system (T6SS) genes like *tssF, tssC,* and *tssE* were examined by qRT-PCR. (**B**) Expression profiles of several genes encoding the complex I of the electron transport chain, including *nuoG, nuoE,* and *nuoC* were examined by qRT-PCR. (**C**) Expression profiles of glyoxylate cycle genes malate synthase and isocitrate lyase, and TCA cycle gene isocitrate dehydrogenase. Each bar represents the mean values of three independent biological replicates along with the SD. Statistical significance was determined by one-way ANOVA. *, *P* ≤ 0.05; **, *P* ≤ 0.001; ***, *P* ≤ 0.001; ****, *P* ≤ 0.0001; ns, non-significant. Tukey’s test was used as a *post hoc* test to determine the statistical significance of all pairs of data.

The increase in the frequency of persistence in Δ*hfq* compared with the WT could be attributed to the differential transcript levels of genes that incur an increased energetic load. Hence, we wanted to check whether the differentially transcribed log-phase genes uncovered from our transcriptome data were also differentially transcribed in the persisters. Subsequently, we isolated total RNA from the spontaneous persisters of WT, Δ*hfq,* and *c-hfq* cells 24 h post-cefepime treatment. Next, using quantitative reverse transcriptase PCR (qRT-PCR), we checked the levels of genes belonging to various classes that showed differential expression in the transcriptome data. We observed that the structural genes of the multicomponent T6SS and T2SS (38, 41) were upregulated in the Δ*hfq* cells (Fig. S7; Fig. 4A). Expression of multicomponent assemblies like the T6SS is known to induce a higher energetic cost (37, 38) and consequently may act as a contributing factor toward the lowered cellular energy levels in Δ*hfq* cells.

Transfer of electrons across the membrane is a key factor in establishing the PMF and, in turn, in ATP synthesis (35). The Δ*hfq* possessed reduced intracellular ATP levels along with downregulation of transcripts involved in electron transfer. This led us to investigate whether there is a differential transcription of electron transport genes among the cefepime persisters of WT, Δ*hfq,* and *c-hfq*. Transcript levels of the *nuo* genes (complex I of the ETC) were significantly reduced (Fig. 4B; Fig. S8). In contrast, genes of the glyoxylate pathway were upregulated in the Δ*hfq* cefepime persisters (Fig. 4C; Fig. S9), suggesting a metabolic shift. This differential regulation likely imposes an energetic burden and lowered reducing equivalents like NADH, contributing to cefepime persistence in *A. baumannii*.

### Alterations in the RNA-binding proximal face of Hfq result in intermediate cefepime persistence

**Fig 5:**
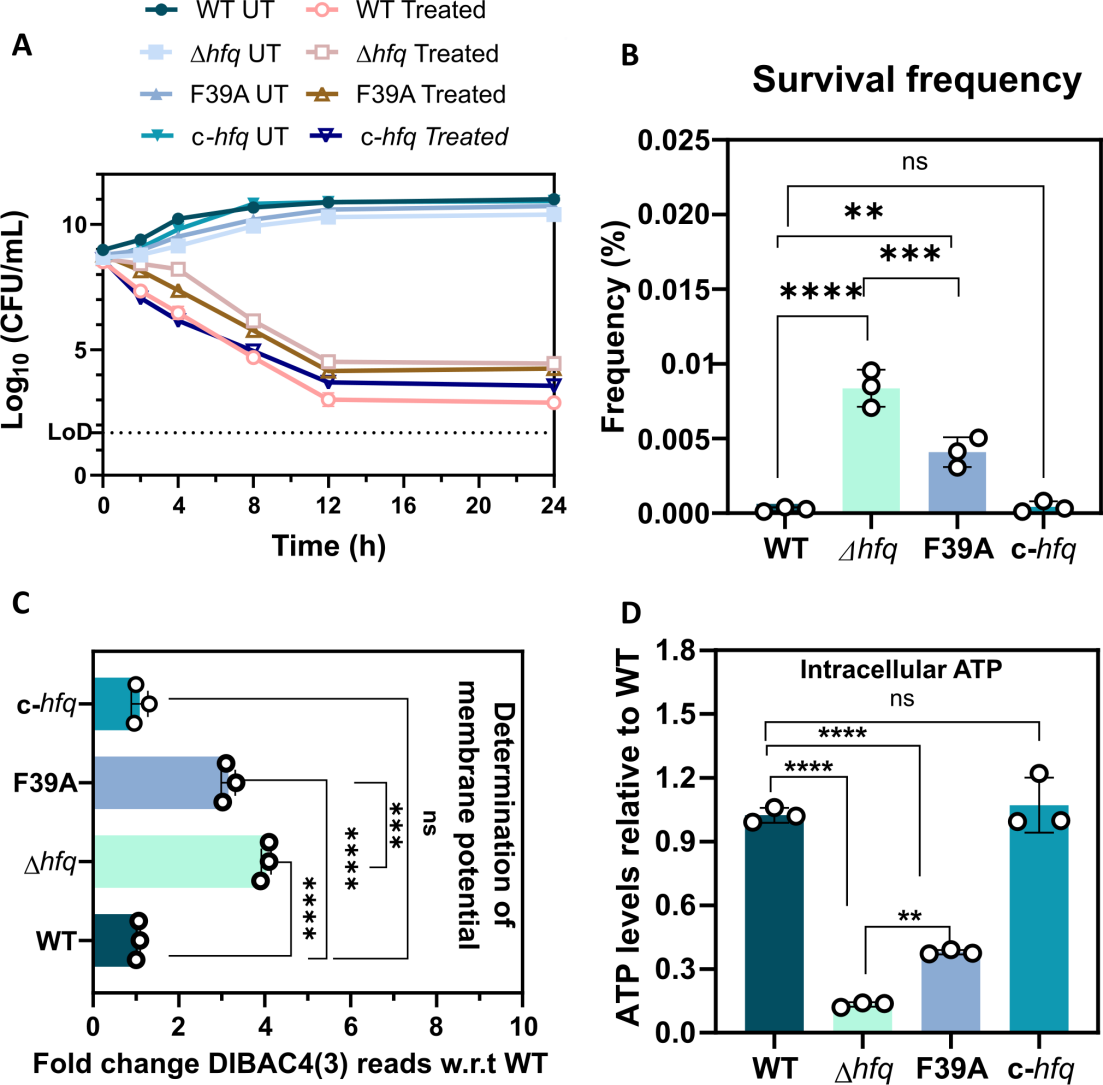
Perturbation of RNA-binding proximal face of *A. baumannii* Hfq impacts its ability to persist under cefepime stress. (**A**) Bi-phasic kill-curve of *A. baumannii* wild-type (WT), Hfq mutant (Δ*hfq*), proximal face mutant (Hfq_F39A_), and complemented strain (c-*hfq*) treated with cefepime at 50× MIC (400 mg/L). Each data point represents three values with SD. The bi-phasic kill-curve is one representative plot of three independent biological replicates. (**B**) Frequency % of persister formation in response to 50× MIC of cefepime (400 mg/L) calculated as (colonies formed in persister at 24 h/no. of colonies of untreated log phase cells at 0 h) × 100. (**C**) Quantification of the degree of membrane depolarization in the cefepime-induced spontaneous persisters of *A. baumannii* WT and Hfq variants grown in LB broth. Measurement of the membrane potential component Δψ was performed using the membrane potential–sensitive fluorescent probe DiBAC4. (**D**) Reduction in intracellular ATP levels in the cefepime-induced spontaneous persisters of *A. baumannii* WT and Hfq variants grown in LB broth using a luciferase/luciferin assay. Each bar represents the mean of three experiments, and the error bars represent the SD. Statistical significance was determined by one-way ANOVA. **, *P* ≤ 0.001; ***, *P* ≤ 0.001; ****, *P* ≤ 0.0001. Tukey’s test was used as a *post hoc* test to determine the statistical significance of all pairs of data.

The RNA chaperone Hfq is known to bring about interaction between sRNA and their cognate mRNA targets via the highly conserved Sm-domain (19, 22). The Sm-domain of the Hfq homohexamer comprises three unique finding faces, the proximal, the distal, and the rim (Fig. S10A) (19). The proximal face of Hfq binds to 3′ poly-U tails of small regulatory RNAs (sRNAs), in turn bringing about post-transcriptional regulation of cognate mRNA targets (42). Earlier, it was reported that an alteration in the amino acid composition of the proximal face of Hfq can lead to an intermediate growth fitness when compared with WT and Δ*hfq* cells (43). Importantly, targeting *E. coli* Hfq using a cyclic peptide resulted in an intermediate sensitivity of the WT-treated cells compared with the untreated and *hfq* deletion mutant strains when exposed to novobiocin (8). Hence, we modified an RNA-binding face residue on the proximal face of Hfq to check whether such modification results in altered growth and impacts antibiotic persistence. As per previous reports in *E. coli*, the proximal face phenylalanine at the 39th position of the Hfq protein (F39), when mutated to alanine (F39A), results in a loss of interaction between a wide range of sRNA-mRNA interactions native to *E. coli* (22). Hence, we cloned and confirmed a Hfq_F39A_ mutant genetic construct and introduced it into the chromosome of *A. baumannii* Δ*hfq* cells using a homologous recombination-based strategy (Fig. S1). We found that this strain also exhibits an intermediate growth defect in LB (Fig. S10A and B) and an intermediate cefepime persister frequency compared with WT and Δ*hfq* (Fig. 5A and B). To further investigate the underlying cause, we quantified the intracellular ATP along with the membrane polarization status of cefepime persisters of WT, Δ*hfq*, Hfq_F39A_, and c-*hfq* cells. Our results show that the persister cells of Δhfq exhibit an increased membrane depolarization accompanied by significantly lowered intracellular ATP levels, whereas the Hfq_F39A_ persisters exhibited an intermediate perturbation of these parameters (Fig. 5C and D). Additionally, the ATP levels and membrane polarization status of the WT and the c-*hfq* strains were similar, indicating that chromosomal complementation restores cellular energetics in cefepime persisters. Our findings show a correlation between perturbed cellular energetics and increased persistence imparted by the loss of *hfq*. Multiple studies have pointed out that treating bacterial cells from different genera with Carbonyl cyanide m-chlorophenyl hydrazine (CCCP) resulted in an increased persistence phenotype due to perturbed metabolism of the cell (44–46). Hence, to determine whether disruption of cellular energetics influences the bactericidal activity of cefepime, we exposed WT cells to a sub-inhibitory concentration of CCCP and subsequently performed a time-kill assay in the presence of cefepime. Indeed, the WT cells treated with CCCP exhibited enhanced survival post-CCCP exposure (Fig. S10C). Hence, we can conclude that the overall decrease in cellular energy reserve resulting from the loss or perturbation of *hfq* contributes toward the increased persister frequency in *A. baumannii*.

### Loss of *hfq* results in attenuated virulence while enhancing *in vivo* tolerance against cefepime

**Fig 6:**
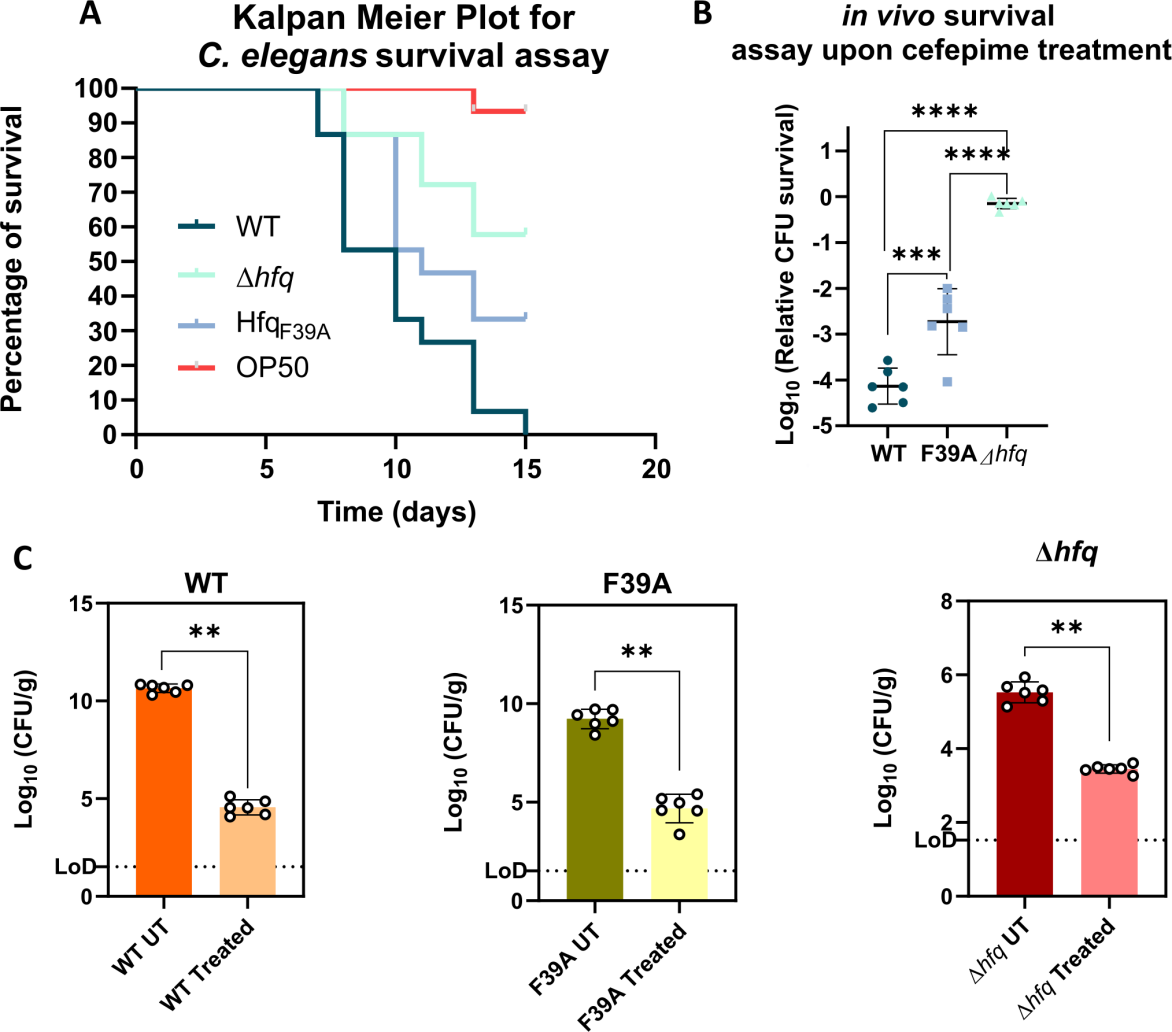
Assessment of the virulence potential of the *A. baumannii* Hfq mutants in comparison to the wild-type. (**A**) Kaplan Meier plot representing the survival of immunosuppressed *C. elegans* worms (strain AU37) feeding on WT *A. baumannii* and *A. baumannii* Δ*hfq* and Hfq_F39A_ and *E. coli* OP50 (negative control) strains. The plot represents one of the three biological replicates with 15 worms in each replicate. (**B**) The percentage of relative CFU survival of bacterial burden from murine lung upon treating different *A. baumannii* strains with cefepime. The relative CFU survival is calculated using equation 1. The error bars represent SD. Statistical significance was determined by one-way ANOVA (*, *P* ≤ 0.05; **, *P* ≤ 0.001; ****, *P* ≤ 0.0001; ns, non-significant). Tukey’s test was used as a *post hoc* test to determine the statistical significance of all pairs of data. (**C**) Organ load in lungs of untreated (UT) and treated individual mice infected with different strains of *A. baumannii*. The error bars represent SD. Statistical significance was determined by Mann-Whitney’s unpaired *t*-test (non-parametric), and the *P*-value was determined to be ≤0.001.

The deletion of *hfq* has been attributed to loss of virulence in *A. baumannii* and other gram-negative pathogens (18). The overall attenuation of pathophysiology has made the Hfq protein and its regulatory networks an interesting area for developing new drugs (8). As the Δhfq and Hfq_F39A_ exhibits severe to mild growth defects, respectively, we sought to explore whether these strains exhibit attenuation of survival fitness when exposed to isolated infection-relevant conditions. We observed that ~26% of the WT cells survive in the presence of human blood-derived neutrophils compared to the 4.25% and ~8.7% survival rates of the Δ*hfq* and Hfq_F39A_ strains, respectively (Fig. S11A). This observation confirms that loss of *hfq* renders the pathogenic *A. baumannii* more prone to neutrophil-mediated killing. To further assess whether deletion of *hfq* or mutation in its RNA-binding residue may impact the virulence of *A. baumannii,* we assessed mortality in immunosuppressed *C. elegans* when fed with the WT, Δ*hfq,* and Hfq_F39A_ strains of this pathogen (Fig. 6A). We observed that over the course of 15 days of feeding, the WT cells caused ~100% mortality in *C. elegans*, whereas the Δ*hfq* and Hfq_F39A_ resulted in ~30% and ~65% mortality, respectively (Fig. S11B). Consequently, loss of Hfq or disruptions to its RNA-binding face dysregulate multiple genetic networks, resulting in significantly reduced virulence.

These findings point to the dichotomous role of Hfq in *A. baumannii*, as its loss results in an attenuation of virulence with an increased ability to persist under antibiotic stress. Therefore, we wanted to understand how *A. baumannii* Δ*hfq* may respond to cefepime treatment during a course of infection in an *in vivo* pneumonia model. We hypothesized that as deletion of *hfq* confers a survival advantage in *A. baumannii* when exposed to cefepime, this should allow better relative survival of the *hfq* deletion mutant compared with the WT inside its host. As a proof of concept, we assessed the efficacy of cefepime in an immunocompromised murine-lung infection model against *A. baumannii* WT, Δ*hfq,* and Hfq_F39A_ as described in the schematic Fig. S11C. In recent studies, enumeration of *in vivo* survival helped determine the relevance of *in vitro* assays in murine models (47, 48), highlighting the importance of this approach. Consequently, we plotted the relative CFU survival using Equation 1 (Fig. S11C) to understand how well the mutants survive cefepime treatment *in vivo*. Our results show that the WT cells exhibit significantly reduced relative survival compared to the Hfq_F39A_ and Δhfq cells (Fig. 6B), with the latter showing the highest relative survival. However, these observations do not provide a clear picture of the influence of cefepime on *A. baumannii* WT and Hfq mutant strains. We observed that the overall organ load was reduced in the case of Δ*hfq* compared with WT in both treated and untreated populations (Fig. 6C). Importantly, this trend was also reflected post-cefpime treatment in the lungs. These observations prove that although deletion or alteration of Hfq increases *in vitro* antibiotic persistence and *in vivo* relative survival, their inherent loss in virulence results in lowered CFU counts *in vivo*. Hence, in a clinical setting, targeting Hfq is still of relevance, as it results in an overall reduction in CFU count post-cefepime treatment inside a murine lung.

## DISCUSSION

Antibiotic persistence occurs when a subpopulation of bacterial cells withstands exposure to lethal concentrations of antibiotics, leading to recurrent infections and treatment failures (2). Although persistence is a non-inheritable trait, repeated unsuccessful antibiotic exposure can promote the selection of drug-resistant variants (6). This phenotype results from multiple underlying mechanisms (1, 7), with recent studies identifying global regulatory networks, such as post-transcriptional regulation, as key factors in establishing antibiotic persistence (7). Researchers have explored the roles of RNA chaperones like Hfq, ProQ, and small RNAs (sRNAs) in conferring persistence to different bacterial species (7). As discussed earlier, deletion of *hfq* does not equate to a universal loss or gain in antibiotic persistence phenotype, as in *E. coli,* it leads to increased persistence, whereas the opposite has been reported in *Yersinia ruckeri* and *Aeromonas veronii* (9–11). Another RNA chaperone, ProQ, was recently implicated in the antibiotic persistence phenotype of *Salmonella* spp. (12) Consequently, it was imperative to investigate the contribution of *A. baumannii*’s Hfq toward persistence under antibiotic stress.

**Fig 7:**
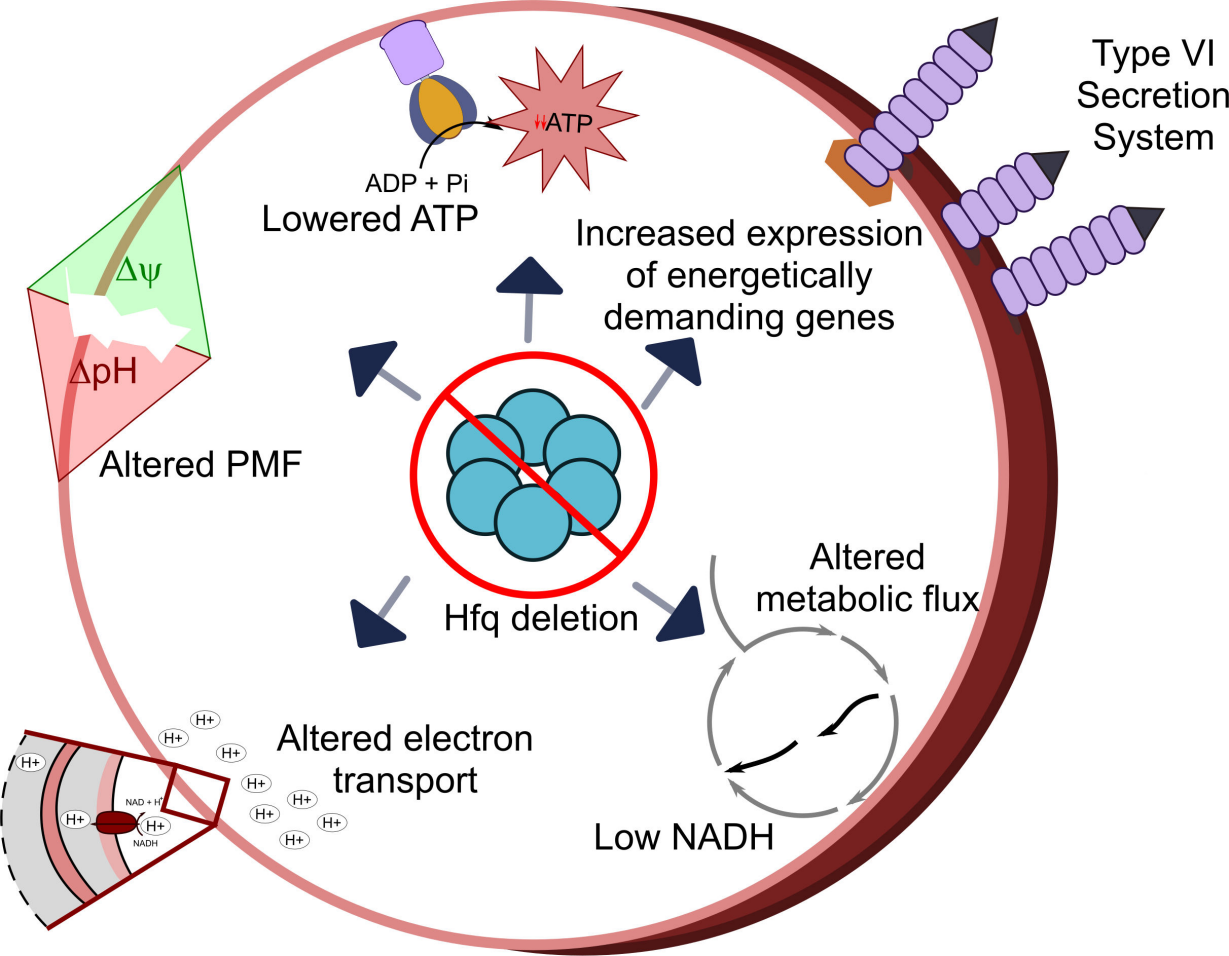
Pleiotropic dysregulation of physiological fitness resulting from deletion of *hfq* imparts enhanced antibiotic persistence in *A. baumannii*. The deletion of *hfq*(Δ*hfq*) causes the upregulation of energetically demanding genes like those of the type II and type VI secretion systems and alters metabolic flux by upregulating the genes of the glyoxylate shunt. Furthermore, the *hfq* deletion mutant strains of *A. baumannii* exhibit perturbed proton motive force (PMF) and reduced intracellular NADH and ATP levels. These factors cumulatively result in an enhanced propensity of the Δ*hfq* strains to form spontaneous persister cells that allow them to survive under a multitude of antibiotics, especially cefepime, with a concomitant loss of virulence.

The findings of this study reveal the dual and contrasting roles of the RNA chaperone Hfq in *A. baumannii* and demonstrate its critical involvement in both virulence and antibiotic persistence. The role of Hfq in post-transcriptional regulation is well established, whereby Hfq regulates protein expression from mRNAs by mediating their robust interactions with cognate sRNA at the post-transcriptional level (19). In view of this, our results demonstrate that deletion of *hfq* disrupts cellular energetics, reduces intracellular ATP levels, and perturbs proton motive force (PMF) by altering the mRNA transcript levels of these genes in *A. baumannii* (Fig. 7). Collectively, these perturbations impose an energetic burden on the cell (Fig. 7). Energetic and metabolic disruptions lead to growth defects but simultaneously enhance the frequency of spontaneous persisters, particularly under cefepime stress. Additionally, we found that disruption of cellular energetics using CCCP increased the persister frequency of WT *A. baumannii* against cefepime, further linking the Δ*hfq*-induced energetic burden with increased persistence. Next, to better understand how a drug that partially inhibits *hfq* activity, we introduced an Hfq variant with a mutation in its proximal RNA-binding face (Hfq_F39A_). Time-kill and cellular energetic profiling revealed that Hfq_F39A_ has an intermediate effect on persistence, further implicating Hfq in balancing cellular energetics and antibiotic persistence. Furthermore, although Δ*hfq* cells exhibited diminished virulence—rendering them more susceptible to neutrophil-mediated killing and less pathogenic in infection models—they paradoxically showed enhanced survival under cefepime treatment during lung infections. The *hfq* mutants exhibited reduced virulence compared with WT (Fig. 6C); however, they better resisted clearance by cefepime (Fig. 6B). Our findings indicate that loss or disruption of Hfq reduces *A. baumannii* virulence, concomitantly increasing antibiotic persistence, particularly conferring the ability to persist in the presence of first-line antibiotics like cefepime (27). This further highlights the physiological importance of *A. baumannii* Hfq in antibiotic persistence when inside its host.

Deletion of *hfq* impairs *A. baumannii*’s virulence by disrupting key physiological functions (16, 25), making this a potential drug target, particularly for combinatorial therapies. Previously, El-Mowafi et al. demonstrated that the cyclic peptide RI20 inhibits Hfq, thereby enhancing the sensitivity of *E. coli* to antibiotics (8). Infections caused by *A. baumannii* are particularly difficult to treat due to the disrupted immune responses in immunocompromised patients. As deletion of *hfq* renders this pathogen less virulent in immunocompromised hosts (Fig. 6C) (16, 25), it has emerged as a promising alternative drug target in *A. baumannii*, especially given the WHO-assigned critical priority status of this pathogen (14). However, pathogenic bacteria employ a wide array of approaches, including antibiotic persistence, to survive lethal antibiotic doses (1). Findings from our study shed light on the fact that targeting Hfq via combinatorial strategies may induce increased antibiotic persistence in pathogenic *A. baumannii*. This is especially true, given the high degree of conservation of the Hfq protein among different *A. baumannii* strains (25). Therefore, results from this study are crucial for developing strategies to target this protein in the future.

The Hfq protein plays a crucial role in maintaining cellular physiology in bacterial pathogens, and its importance is well-established (18). Loss of *hfq* disrupts post-transcriptional regulatory circuits within the cell (18), and in *Acinetobacter* species, the Hfq protein exhibits a high degree of conservation (25). In different *Acinetobacter sp*., genes related to amino acid metabolism, biofilm formation, virulence, and drug efflux are reported to be under post-transcriptional regulation (49–52). Additionally, results from our study indicate the probable contribution of Hfq in the intrinsic antibiotic resistance of *A. baumannii* (Table S4), and earlier studies demonstrate that *hfq* deletion leads to a 2-fold to 4-fold decrease in MIC values against various antibiotics in different bacterial pathogens (16, 26). However, the endogenous post-transcriptional pathways contributing to this remain largely unexamined in *A. baumannii*. An important aspect of our study is that it has unveiled the influence of *hfq* deletion on the mRNA landscape of *A. baumannii* and linked perturbed cellular energetics to antibiotic persistence in *hfq* mutants of this pathogen. A key finding of this study was the increased transcription of genes of the glyoxylate shunt along with a concomitant perturbation of TCA-cycle transcript levels upon hfq deletion. To date, there are no direct reports of Hfq-mediated post-transcriptional regulation of the glyoxylate shunt in bacteria. However, the role of Hfq in regulating central metabolic pathways and bringing about carbon catabolite repression (CCR) has been established in different bacterial species (53–55). In *E. coli,* for example, an Hfq-dependent sRNA called Spot 42 is involved in establishing a non-coding arm of CCR. Spot 42 is expressed in glucose-rich conditions and represses expression from mRNA encoding non-essential carbon source-utilizing proteins (55). Another such example can be found in *Salmonella enterica,* where an sRNA SgrS plays an important role in carbohydrate uptake and utilization (55). In *Pseudomonas sp.,* CCR is established via Hfq and another partner protein called catabolite repression control protein (Crc) at the post-transcriptional level (53). Moreover, Hfq-Crc-mediated post-transcriptional regulation has also been linked to antibiotic susceptibility in *Pseudomonas sp*. (56). Hence, the observed upregulation of glyoxylate shunt genes upon *hfq* deletion may have an underlying non-coding sRNA-mediated regulation that remains to be deciphered. Given the established relevance of glyoxylate shunt in antibiotic tolerance and resistance (57, 58), its perturbation upon deletion of Hfq is of significance. Our recent findings have demonstrated that non-coding regulatory arms impact expression of virulence-associated T6SS in *A. baumannii* (36). Hence, building upon our observations, future studies will help decipher key sRNA players of Hfq-driven regulation of metabolic pathways in pathogens like *A. baumannii*. Therefore, this work emphasizes the potential for further exploration of endogenous post-transcriptional networks in *A. baumannii* through techniques like RNA interaction by ligation and sequencing (RIL-seq) (23, 59, 60) under various stress conditions. Studying endogenous Hfq-mediated sRNA networks will highlight the probable reasons behind the altered transcriptome in the *hfq* deletion mutant of *A. baumannii*. Consequently, findings from this study will pave the way for a deeper fundamental analysis of the post-transcriptional regulatory landscape in this pathogen.

Overall, this work provides valuable insights into the complex role of Hfq in modulating *A. baumannii* pathophysiology. Additionally, this study offers a foundation for future research into post-transcriptional regulatory mechanisms of this pathogen. Importantly, we found that deleting *A. baumannii* Hfq could lead to enhanced survival in the presence of antibiotics both *in vitro* and *in vivo*. However, owing to its diminished virulence, Δ*hfq* strains exhibit lower organ burden postantibiotic treatment. This caveat highlights the fact that targeting Hfq still holds clinical relevance as it leads to significant attenuation of virulence. Moreover, results from our *in vivo* assay point to the fact that straightforward *in vitro* assays of antibiotic persistence may not always give a complete picture of the physiological relevance of this phenotype. Henceforth, investigators may focus on unraveling the intricate sRNA-mRNA networks mediated by Hfq and exploring strategies to minimize the risk of increased persistence while leveraging its attenuation of virulence for effective treatment options.

## MATERIALS AND METHODS

### Bacterial strains, culture conditions, and reagents

The strains *A. baumannii* ATCC 17978 VU and *E. coli* HST08 (Takara) were used for the study. A detailed list of strains is listed in Table S1. The growth medium was Luria Bertani (LB) broth (Himedia), agar (Himedia), or Leeds Acinetobacter agar (Himedia). Antibiotics used in this study were procured from Sigma-Aldrich and Tokyo Chemicals Industries Limited (TCI) and stored as prescribed. DiBAC4(3) was purchased from Thermo Fisher Scientific, BacTiter-Glo for ATP enumeration was procured from Promega. Resazurin and MTT were procured from Sigma and Biobasic, respectively. The list of plasmids and DNA oligos (primers) used in this study is listed in Tables S2 and S3, respectively.

### Determination of minimum inhibitory concentration

Serial 2-fold dilutions of each compound were prepared in LB and added to 96-well plates, as per Clinical & Laboratory Standards Institute (CLSI) guidelines. Overnight-grown cultures of test strains were sub-cultured in sterile tubes at 37°C and 180 RPM until they reached an optical density (OD) of 0.4 at 600 nm. Cultures were subsequently diluted to achieve an inoculum density of ~105 CFU/mL and added to plates containing the compound dilutions. Plates were incubated for 16 hours at 37°C in a static incubator. At the end of incubation, absorbance was recorded on a plate reader (SpectraMax iD5) at 600 nm.

### Multimode plate reader-based growth assessment

Overnight cultures of *A. baumannii* in LB broth were allowed to reach OD600 = 0.5–0.7, and subsequently, the OD600 was adjusted to 0.4. This culture was subsequently diluted 1,000 times in fresh LB broth, and 200 µL of this diluted inoculum was dispensed into sterile clear-bottom 96-well plates (Genaxy). The plate is then kept in a multimode plate reader with lid (Synergy H1, Biotek) pre-set at 37°C, and OD600 is measured at desired time intervals with shaking. The OD600 vs Time is plotted as an *XY* curve in GraphPad (Prism 8) software.

### Recombinant DNA and chromosomal complementation procedures

Chromosomal complementation of Hfq was created using a homologous recombination-based approach as described by Dubey et al. (61). In brief, a nucleic acid sequence of the Hfq coding region along with its 5′ UTR was amplified from *A. baumannii* ATCC17978 VU genomic DNA. The PCR product was subsequently cloned in a pUC18 vector, followed by an apramycin-FRT cassette. Next, the downstream 500 bp region of *hfq* on its 3′ end was cloned after the apramycin-resistant marker. Next, this pUC18 construct harboring the wild-type *hfq* was used to introduce an F39A mutation into the Hfq CDS using inverse PCR and subsequent ligation by InFusion cloning (Takara). The ligation product was transformed into an *E. coli* HST08 cell, and transformants were selected on LB agar plates supplemented with apramycin (20 mg/L). The constructs for Hfq (*c-hfq* and *hfqF39A*) variants were confirmed by Sanger sequencing. A PCR amplicon from these clones was used for chromosomal complementation of the *hfq*(*c-hfq* and *hfqF39A*) genes into the native *hfq* chromosomal locus of an *A. baumannii* Δ*hfq* strain. The strains were evaluated for polar effect using qPCR, and it was found that the chromosomal complementation does not affect upstream or downstream genes (data not shown). It should be noted that reintroducing a gene in its original chromosomal locus might induce a polar effect through scar sequences at the post-transcriptional level. However, in addition to the qRT-PCR data, Hfq complement strain did not exhibit any phenotypic defect (e.g., growth phenotype) compared with the WT. Together, these observations point to the fact that there was no polar effect due to chromosomal complementation. Methods pertaining to chromosomal complementation are described in Supplemental File section S1.

### Persister and non-heritability assay

A single colony of *A. baumannii* variants was cultured separately at 37°C for 16  h in LB, diluted 1:100 in fresh medium, and incubated until mid-log phase OD600 0.6–0.7 and adjusted to 0.5. These cultures were treated with 50× or 100× MIC of antibiotics for 24 h at 37°C in shaking conditions. CFU count was enumerated by harvesting the antibiotic-treated and untreated cells at different time points (0, 2, 4, 8, and 24  h) and serially diluting and spreading them on LB agar plates. From the 24 h treated CFUs, two colonies were re-inoculated into fresh LB medium, and an *in vitro* susceptibility experiment was performed to ensure the MIC had not changed in order to confirm that the surviving colonies were persister cells. To assess whether perturbation of cellular metabolism arising from disrupted PMF and ATP levels, the CCCP-exposed persister assay was carried out; a detailed protocol is given in the Supplemental File, section S2. To assess whether the persister phenotype is inheritable, the persister assay was repeated thrice, and percentage survival or frequency % was determined ([colonies formed in persister at 24 h/No. of colonies of untreated Log phase cells at 0 h) × 100]*.* A detailed protocol is given in the Supplemental File, section S3.

### Assays for measuring ATP levels, membrane polarization, and NADH/H+ levels

All the different strains of WT and Hfq mutants of *A. baumannii* were subjected to different dye-based assays. A detailed protocol for each of these assays is described in the Supplemental File, section S4.

### Quantification of RNA transcripts using quantitative reverse-transcriptase PCR (qRT-PCR) in Cefepime persisters

Persisters of *A. baumannii* WT Δ*hfq and c-hfq* cells were generated using the strategy described above. At 24 h, the cells were harvested and washed twice in sterile 1× PBS. After washing the cell pellet with 1× PBS, RNA was extracted from the bacterial cells using the classic phenol-chloroform method, followed by DNase treatment. cDNA synthesis was performed using the PrimeScript 1st strand cDNA Synthesis Kit (TakaRa) according to the manufacturer’s instructions. Amplifications were achieved using a 3-step program on a QuantStudio five system (Thermo Scientific). Transcript abundance was calculated using the ΔΔC_T_ method and normalized by the 16S gene.

### RNA-sequencing data analysis

RNA was isolated from OD600 = 0.5 cultures of WT and Δhfq cells of *A. baumannii* grown in LB broth with shaking at 180 rpm in a 5 mL culture. In brief, the cells were harvested and washed twice in sterile 1× PBS. After washing the cell pellet with 1× PBS, RNA was extracted from the bacterial cells using the classic phenol-chloroform method. The extracted RNA was subjected to DNase treatment, following which the RNA samples were precipitated using sodium acetate precipitation. RNA sequencing and differential transcript level analysis were performed by miBiome Therapeutics (Mumbai, India) using the Illumina NovaSeq 6000 platform (Illumina) using a PE150 chemistry. Before RNA-seq, the integrity and concentration of total RNA were determined using an Agilent 2100 Bioanalyzer system in combination with an RNA 6000 Nano kit (Agilent). rRNA was depleted using the Ribo-Zero rRNA removal kit (for bacteria) (Illumina), and paired-end cDNA libraries were prepared. The quality of the reads was assessed using FastQC v 0.11.3 before proceeding with the downstream analysis. The reads were trimmed using Fastp v0.20.1 when the quality of the bases dropped below 30, and length was less than 50 bp, and adapters were removed. Reads were rechecked for improvement in quality before proceeding further. A transcriptome index was prepared using the DNA of reference assembly *A. baumannii* ATCC17978 VU genome (NCBI: CP018664.1) (36). The quality-passed reads were mapped onto the reference transcriptome using bowtie2 and were quantified with featureCounts v2.0.1 using gff3 file of the reference genome. The DESeq2 was used for differential expression analysis. Only genes with read count sum >10 in all the samples were analyzed further. After DESeq2, genes were filtered based on the false discovery rate cutoff (FDR) ≤ 0.05 and minimum expression fold change (FC) ≥2.

### Estimation of cell survival from phagocytosis

Blood samples were collected from healthy human male and female volunteers, as per the approved guidelines at the Institute hospital. Neutrophils were isolated using Polymorphprep (ProteoGenix), as per the manufacturer’s protocol. The estimation of bacterial survival from phagocytic cell-mediated killing was performed as described previously (62). The detailed procedure is provided in the Supplemental File, section S5.

### *Caenorhabditis elegans* survival assay for virulence studies

Immunosuppressed strain AU37 was used for the survival assay. Worm maintenance and egg preparation were done as per worm book guidelines. The assay was performed in sterile 6-well polystyrene plates (Tarsons) with each *A. baumannii* mutant, that is, WT, Hfq_F39A_, and Δ*hfq* spotted in biological triplicate, and 15 L4-young adult worms were transferred to each well and incubated at 25°C. The worms were transferred to fresh medium plates along with the respective *A. baumannii* mutant after 72 h to avoid any fungal contamination. The worms that did not respond to touch and displayed no movement were considered dead. The number of surviving worms each day was recorded for 15 days.

### *A. baumannii* murine pneumonia model to assess virulence

All animal experiments under protocol BT/IAEC/2017/01 were reviewed and approved by the Institute Animal Ethics Committee of the Indian Institute of Technology Roorkee. A detailed protocol of the experiment is mentioned in the Supplemental File, section S6. The MIC of the *A. baumannii* strains isolated from the organs was determined, which was found to remain unchanged. Relative clearance percentage was determined using equation 1:


(1) $$Relative\;CFU\;survival=\left\{\left(\frac{organ\;load\;in\;CFU\;enumerated\;in\;treated\;individual\;animals\;}{mean\;\;of\;organ\;burden\;in\;CFU\;enumerated\;in\;untreated\;group\;\left(n=6\right)}\;\right)\times 100\right\}\%.$$

### Statistical analysis

Statistical analyses were carried out using Prism 8 software (GraphPad, CA, USA). One-way analysis of variance (ANOVA) with Tukey’s multiple-comparison *post hoc* test was used for comparisons between three or more conditions. *P*-values are indicated as follows: *, *P* ≤ 0.05; **, *P* ≤ 0.001; ***, *P* ≤ 0.001; ****, *P* ≤ 0.0001; ns, non-significant. For comparison between two events, Mann-Whitney’s unpaired *t*-test (non-parametric) was applied, and the *P*-value was determined to be *P* ≤ 0.001. 

## Data Availability

Raw RNA-sequence data have been deposited at NCBI Sequence Read Archive (NCBI SRA) with accession number PRJNA81219993. All other data are available upon request from the corresponding author.

## SUPPLEMENTAL MATERIAL

Data S1 msystems.00320-25-s0001.xlsx Significantly differentially expressed genes between wild-type and *hfq* deletion mutant *A. baumannii* cells.

Supplemental File msystems.00320-25-s0002.docx Additional experimental details and supplemental figures and tables.

ASM does not own the copyrights to Supplemental Material that may be linked to, or accessed through, an article. The authors have granted ASM a non-exclusive, world-wide license to publish the Supplemental Material files. Please contact the corresponding author directly for reuse.

## References

[1] Huemer M, Mairpady Shambat S, Brugger SD, Zinkernagel AS. 2020. Antibiotic resistance and persistence-Implications for human health and treatment perspectives. EMBO Rep 21:e51034. doi:10.15252/embr.202051034 33400359 PMC7726816

[2] Balaban NQ, Helaine S, Lewis K, Ackermann M, Aldridge B, Andersson DI, Brynildsen MP, Bumann D, Camilli A, Collins JJ, Dehio C, Fortune S, Ghigo J-M, Hardt W-D, Harms A, Heinemann M, Hung DT, Jenal U, Levin BR, Michiels J, Storz G, Tan M-W, Tenson T, Van Melderen L, Zinkernagel A. 2019. Publisher Correction: Definitions and guidelines for research on antibiotic persistence. Nat Rev Microbiol 17:460–460. doi:10.1038/s41579-019-0207-4 31036919 PMC7609342

[3] Balaban NQ, Merrin J, Chait R, Kowalik L, Leibler S. 2004. Bacterial persistence as a phenotypic switch. Science 305:1622–1625. doi:10.1126/science.1099390 15308767

[4] Poudel AN, Zhu S, Cooper N, Little P, Tarrant C, Hickman M, Yao G. 2023. The economic burden of antibiotic resistance: a systematic review and meta-analysis. PLoS One 18:e0285170. doi:10.1371/journal.pone.0285170 37155660 PMC10166566

[5] Cosgrove SE, Carmeli Y. 2003. The impact of antimicrobial resistance on health and economic outcomes. Clin Infect Dis 36:1433–1437. doi:10.1086/375081 12766839

[6] Eisenreich W, Rudel T, Heesemann J, Goebel W. 2022. Link between antibiotic persistence and antibiotic resistance in bacterial pathogens. Front Cell Infect Microbiol 12:900848. doi:10.3389/fcimb.2022.900848 35928205 PMC9343593

[7] Sett A, Dubey V, Bhowmik S, Pathania R. 2024. Decoding bacterial persistence: mechanisms and strategies for effective eradication. ACS Infect Dis 10:2525–2539. doi:10.1021/acsinfecdis.4c00270 38940498

[8] El-Mowafi SA, Alumasa JN, Ades SE, Keiler KC. 2014. Cell-based assay to identify inhibitors of the Hfq-sRNA regulatory pathway. Antimicrob Agents Chemother 58:5500–5509. doi:10.1128/AAC.03311-14 25001303 PMC4135888

[9] Kim Y, Wood TK. 2010. Toxins Hha and CspD and small RNA regulator Hfq are involved in persister cell formation through MqsR in Escherichia coli. Biochem Biophys Res Commun 391:209–213. doi:10.1016/j.bbrc.2009.11.033 19909729 PMC2812665

[10] Zhang L, Yu W, Tang Y, Li H, Ma X, Liu Z. 2019. RNA chaperone hfq mediates persistence to multiple antibiotics in Aeromonas veronii. Microb Pathog 132:124–128. doi:10.1016/j.micpath.2019.04.045 31054368

[11] Calderón IL, Barros MJ, Montt F, Gil F, Fuentes JA, Acuña LG. 2021. The RNA chaperone Hfq participates in persistence to multiple antibiotics in the fish pathogen Yersinia ruckeri. Microorganisms 9:1404. doi:10.3390/microorganisms9071404 34209738 PMC8308036

[12] Rizvanovic A, Michaux C, Panza M, Iloglu Z, Helaine S, Wagner EGH, Holmqvist E. 2022. The RNA-binding protein ProQ promotes antibiotic persistence in Salmonella. mBio 13:e02891-22. doi:10.1128/mbio.02891-22 36409088 PMC9765298

[13] Harding CM, Hennon SW, Feldman MF. 2018. Uncovering the mechanisms of Acinetobacter baumannii virulence. Nat Rev Microbiol 16:91–102. doi:10.1038/nrmicro.2017.148 29249812 PMC6571207

[14] WHO. 2024. WHO bacterial priority pathogens list, 2024: bacterial pathogens of public health importance to guide research, development and strategies to prevent and control antimicrobial resistance. World Health Organization, Geneva

[15] Kovacic A, Seruga Music M, Dekic S, Tonkic M, Novak A, Rubic Z, Hrenovic J, Goic-Barisic I. 2017. Transmission and survival of carbapenem-resistant Acinetobacter baumannii outside hospital setting. Int Microbiol 20:165–169. doi:10.2436/20.1501.01.299 29529327

[16] Sharma A, Dubey V, Sharma R, Devnath K, Gupta VK, Akhter J, Bhando T, Verma A, Ambatipudi K, Sarkar M, Pathania R. 2018. The unusual glycine-rich C terminus of the Acinetobacter baumannii RNA chaperone Hfq plays an important role in bacterial physiology. J Biol Chem 293:13377–13388. doi:10.1074/jbc.RA118.002921 30002121 PMC6120210

[17] Kuo HY, Chao HH, Liao PC, Hsu L, Chang KC, Tung CH, Chen CH, Liou ML. 2017. Functional characterization of Acinetobacter baumannii lacking the RNA chaperone Hfq. Front Microbiol 8:2068. doi:10.3389/fmicb.2017.02068 29163381 PMC5663733

[18] Chao Y, Vogel J. 2010. The role of Hfq in bacterial pathogens. Curr Opin Microbiol 13:24–33. doi:10.1016/j.mib.2010.01.001 20080057

[19] Vogel J, Luisi BF. 2011. Hfq and its constellation of RNA. Nat Rev Microbiol 9:578–589. doi:10.1038/nrmicro2615 21760622 PMC4615618

[20] Rodgers ML, O’Brien B, Woodson SA. 2023. Small RNAs and Hfq capture unfolded RNA target sites during transcription. Mol Cell 83:1489–1501. doi:10.1016/j.molcel.2023.04.003 37116495 PMC10176597

[21] Panja S, Woodson SA. 2012. Hexamer to monomer equilibrium of E. coli Hfq in solution and its impact on RNA annealing. J Mol Biol 417:406–412. doi:10.1016/j.jmb.2012.02.009 22326348 PMC3303956

[22] Zhang A, Schu DJ, Tjaden BC, Storz G, Gottesman S. 2013. Mutations in interaction surfaces differentially impact E. coli Hfq association with small RNAs and their mRNA targets. J Mol Biol 425:3678–3697. doi:10.1016/j.jmb.2013.01.006 23318956 PMC3640674

[23] Melamed S, Adams PP, Zhang A, Zhang H, Storz G. 2020. RNA-RNA interactomes of ProQ and Hfq reveal overlapping and competing roles. Mol Cell 77:411–425. doi:10.1016/j.molcel.2019.10.022 31761494 PMC6980735

[24] Potts AH, Vakulskas CA, Pannuri A, Yakhnin H, Babitzke P, Romeo T. 2017. Global role of the bacterial post-transcriptional regulator CsrA revealed by integrated transcriptomics. Nat Commun 8:1596. doi:10.1038/s41467-017-01613-1 29150605 PMC5694010

[25] Sett A, Maiti PK, Garg K, Hussain A, Saini S, Pandey S, Pathania R. 2024. “GGFGGQ” repeats in Hfq of Acinetobacter baumannii are essential for nutrient utilization and virulence. J Biol Chem 300:107895. doi:10.1016/j.jbc.2024.107895 39424139 PMC11617691

[26] Wang Y, Teng Y, Geng J, Long J, Yang H, Duan G, Chen S. 2023. Involvement of RNA chaperone hfq in the regulation of antibiotic resistance and virulence in Shigella sonnei. Res Microbiol 174:104047. doi:10.1016/j.resmic.2023.104047 36868486

[27] Kanafani ZA, Kanj SS. 2024. Acinetobacter infection: treatment and prevention. UpToDate. Available from: https://www.uptodate.com/contents/acinetobacter-infection-treatment-and-prevention

[28] Sittka A, Pfeiffer V, Tedin K, Vogel J. 2007. The RNA chaperone Hfq is essential for the virulence of Salmonella typhimurium. Mol Microbiol 63:193–217. doi:10.1111/j.1365-2958.2006.05489.x 17163975 PMC1810395

[29] Arce-Rodríguez A, Calles B, Nikel PI, de Lorenzo V. 2016. The RNA chaperone Hfq enables the environmental stress tolerance super-phenotype of Pseudomonas putida. Environ Microbiol 18:3309–3326. doi:10.1111/1462-2920.13052 26373442

[30] Saini M, Gaurav A, Hussain A, Pathania R. 2024. Small molecule IITR08367 potentiates antibacterial efficacy of fosfomycin against Acinetobacter baumannii by efflux pump inhibition. ACS Infect Dis 10:1711–1724. doi:10.1021/acsinfecdis.4c00077 38562022

[31] Wos ML, Pollard PC. 2009. Cellular nicotinamide adenine dinucleotide (NADH) as an indicator of bacterial metabolic activity dynamics in activated sludge. Water Sci Technol 60:783–791. doi:10.2166/wst.2009.393 19657174

[32] Saini M, Gaurav A, Kothari A, Omar BJ, Gupta V, Bhattacharjee A, Pathania R. 2023. Small molecule IITR00693 (2-aminoperimidine) synergizes polymyxin B activity against Staphylococcus aureus and Pseudomonas aeruginosa. ACS Infect Dis 9:692–705. doi:10.1021/acsinfecdis.2c00622 36716174

[33] Candeias LP, MacFarlane DPS, McWhinnie SLW, Maidwell NL, Roeschlaub CA, Sammes PG, Whittlesey R. 1998. The catalysed NADH reduction of resazurin to resorufin. J Chem Soc Perkin Trans 2 1:2333–2334. doi:10.1039/a806431h

[34] Grela E, Kozłowska J, Grabowiecka A. 2018. Current methodology of MTT assay in bacteria - a review. Acta Histochem 120:303–311. doi:10.1016/j.acthis.2018.03.007 29606555

[35] Hinkle PC, McCarty RE. 1978. How cells make ATP. Sci Am 238:104–117, doi:10.1038/scientificamerican0378-104 635521

[36] Bhowmik S, Pathak A, Pandey S, Devnath K, Sett A, Jyoti N, Bhando T, Akhter J, Chugh S, Singh R, Sharma TK, Pathania R. 2025. Acinetobacter baumannii represses type VI secretion system through a manganese-dependent small RNA-mediated regulation. mBio 16:e03025-24. doi:10.1128/mbio.03025-24 39704509 PMC11796373

[37] Smith DR, Chapman MR. 2010. Economical evolution: microbes reduce the synthetic cost of extracellular proteins. mBio 1:e00131-10. doi:10.1128/mBio.00131-10 20824102 PMC2932507

[38] Unni R, Pintor KL, Diepold A, Unterweger D. 2022. Presence and absence of type VI secretion systems in bacteria. Microbiology (Reading) 168. doi:10.1099/mic.0.001151 35467500

[39] Dolan SK, Welch M. 2018. The glyoxylate shunt, 60 years on. Annu Rev Microbiol 72:309–330. doi:10.1146/annurev-micro-090817-062257 30200852

[40] Kim BN, Peleg AY, Lodise TP, Lipman J, Li J, Nation R, Paterson DL. 2009. Management of meningitis due to antibiotic-resistant Acinetobacter species. Lancet Infect Dis 9:245–255. doi:10.1016/S1473-3099(09)70055-6 19324297 PMC2760093

[41] Weber BS, Kinsella RL, Harding CM, Feldman MF. 2017. The secrets of Acinetobacter secretion. Trends Microbiol 25:532–545. doi:10.1016/j.tim.2017.01.005 28216293 PMC5474180

[42] Watkins D, Arya D. 2023. Models of Hfq interactions with small non-coding RNA in Gram-negative and Gram-positive bacteria. Front Cell Infect Microbiol 13:1282258. doi:10.3389/fcimb.2023.1282258 37942477 PMC10628458

[43] Ziolkowska K. 2006. Hfq variant with altered RNA binding functions. Nucleic Acids Res 34:709–720. doi:10.1093/nar/gkj464 16449205 PMC1356530

[44] Li M, Sun X, Zhao L, Du W, Shang D. 2024. The antibacterial activity and mechanisms of Trp-containing peptides against multidrug-resistant Pseudomonas aeruginosa persisters. Biochimie 225:133–145. doi:10.1016/j.biochi.2024.05.019 38815647

[45] Grassi L, Di Luca M, Maisetta G, Rinaldi AC, Esin S, Trampuz A, Batoni G. 2017. Generation of persister cells of Pseudomonas aeruginosa and Staphylococcus aureus by chemical treatment and evaluation of their susceptibility to membrane-targeting agents. Front Microbiol 8:1917. doi:10.3389/fmicb.2017.01917 29046671 PMC5632672

[46] Narayanaswamy VP, Keagy LL, Duris K, Wiesmann W, Loughran AJ, Townsend SM, Baker S. 2018. Novel glycopolymer eradicates antibiotic- and CCCP-induced persister cells in Pseudomonas aeruginosa. Front Microbiol 9:1724. doi:10.3389/fmicb.2018.01724 30123191 PMC6085434

[47] Parsons JB, Sidders AE, Velez AZ, Hanson BM, Angeles-Solano M, Ruffin F, Rowe SE, Arias CA, Fowler VG Jr, Thaden JT, Conlon BP. 2024. In-patient evolution of a high-persister Escherichia coli strain with reduced in vivo antibiotic susceptibility. Proc Natl Acad Sci USA 121:e2314514121. doi:10.1073/pnas.2314514121 38190524 PMC10801923

[48] Shook JC, Genito CJ, Darwitz BP, Tyson KJ, Velez AZ, Bridwell SK, Parsons JB, Rowe SE, Marshall CW, Conlon BP, Thurlow LR. 2025. Diabetes potentiates the emergence and expansion of antibiotic resistance. Sci Adv 11:eads1591. doi:10.1126/sciadv.ads1591 39937900 PMC11817934

[49] Schilling D, Findeiss S, Richter AS, Taylor JA, Gerischer U. 2010. The small RNA Aar in Acinetobacter baylyi: a putative regulator of amino acid metabolism. Arch Microbiol 192:691–702. doi:10.1007/s00203-010-0592-6 20559624

[50] Sharma A, Sharma R, Bhattacharyya T, Bhando T, Pathania R. 2017. Fosfomycin resistance in Acinetobacter baumannii is mediated by efflux through a major facilitator superfamily (MFS) transporter-AbaF. J Antimicrob Chemother 72:68–74. doi:10.1093/jac/dkw382 27650185

[51] Álvarez-Fraga L, Rumbo-Feal S, Pérez A, Gómez MJ, Gayoso C, Vallejo JA, Ohneck EJ, Valle J, Actis LA, Beceiro A, Bou G, Poza M. 2017. Global assessment of small RNAs reveals a non-coding transcript involved in biofilm formation and attachment in Acinetobacter baumannii ATCC 17978. PLoS One 12:e0182084. doi:10.1371/journal.pone.0182084 28763494 PMC5538643

[52] Sarshar M, Scribano D, Palamara AT, Ambrosi C, Masotti A. 2022. The Acinetobacter baumannii model can explain the role of small non-coding RNAs as potential mediators of host-pathogen interactions. Front Mol Biosci 9:1088783. doi:10.3389/fmolb.2022.1088783 36619166 PMC9810633

[53] Sonnleitner E, Wulf A, Campagne S, Pei X-Y, Wolfinger MT, Forlani G, Prindl K, Abdou L, Resch A, Allain FH-T, Luisi BF, Urlaub H, Bläsi U. 2018. Interplay between the catabolite repression control protein Crc, Hfq and RNA in Hfq-dependent translational regulation in Pseudomonas aeruginosa. Nucleic Acids Res 46:1470–1485. doi:10.1093/nar/gkx1245 29244160 PMC5815094

[54] Pei XY, Dendooven T, Sonnleitner E, Chen S, Bläsi U, Luisi BF. 2019. Architectural principles for Hfq/Crc-mediated regulation of gene expression. Elife 8:e43158. doi:10.7554/eLife.43158 30758287 PMC6422490

[55] Papenfort K, Melamed S. 2023. Small RNAs, large networks: posttranscriptional regulons in Gram-negative bacteria. Annu Rev Microbiol 77:23–43. doi:10.1146/annurev-micro-041320-025836 36944261

[56] Pusic P, Sonnleitner E, Krennmayr B, Heitzinger DA, Wolfinger MT, Resch A, Bläsi U. 2018. Harnessing metabolic regulation to increase Hfq-dependent antibiotic susceptibility in Pseudomonas aeruginosa. Front Microbiol 9:2709. doi:10.3389/fmicb.2018.02709 30473687 PMC6237836

[57] Ahmad M, Aduru SV, Smith RP, Zhao Z, Lopatkin AJ. 2025. The role of bacterial metabolism in antimicrobial resistance. Nat Rev Microbiol 23:439–454. doi:10.1038/s41579-025-01155-0 39979446 PMC12173792

[58] Meylan S, Porter CBM, Yang JH, Belenky P, Gutierrez A, Lobritz MA, Park J, Kim SH, Moskowitz SM, Collins JJ. 2017. Carbon sources tune antibiotic susceptibility in Pseudomonas aeruginosa via tricarboxylic acid cycle control. Cell Chem Biol 24:195–206. doi:10.1016/j.chembiol.2016.12.015 28111098 PMC5426816

[59] Melamed S, Peer A, Faigenbaum-Romm R, Gatt YE, Reiss N, Bar A, Altuvia Y, Argaman L, Margalit H. 2016. Global mapping of small RNA-target interactions in bacteria. Mol Cell 63:884–897. doi:10.1016/j.molcel.2016.07.026 27588604 PMC5145812

[60] Matera G, Altuvia Y, Gerovac M, El Mouali Y, Margalit H, Vogel J. 2022. Global RNA interactome of Salmonella discovers a 5’ UTR sponge for the MicF small RNA that connects membrane permeability to transport capacity. Mol Cell 82:629–644. doi:10.1016/j.molcel.2021.12.030 35063132

[61] Dubey V, Gupta R, Pathania R. 2023. Targeting superoxide dismutase confers enhanced reactive oxygen species mediated eradication of polymyxin B induced Acinetobacter baumannii persisters. Antimicrob Agents Chemother 95:e02180-20. doi:10.1128/AAC.02180-20 33593839 PMC8092903

[62] Knippel RJ, Wexler AG, Miller JM, Beavers WN, Weiss A, de Crécy-Lagard V, Edmonds KA, Giedroc DP, Skaar EP. 2020. Clostridioides difficile senses and hijacks host heme for incorporation into an oxidative stress defense system. Cell Host Microbe 28:411–421. doi:10.1016/j.chom.2020.05.015 32526159 PMC7486240

