# Supplementary material for: Hfq balances energetic efficiency and antibiotic persistence in Acinetobacter baumannii
Source: mSystems. 2025 Aug 11;10(9):e00320-25. doi: 10.1128/msystems.00320-25 (PMC12455925; doi:10.1128/msystems.00320-25)
Supplement: Supplemental File — Additional experimental details and supplemental figures and tables. [file msystems.00320-25-s0002.docx]

**SUPPLEMENTAL MATERIAL**

**Hfq deletion drives energetic stress, promoting in vivo antibiotic persistence of *Acinetobacter baumannii***

Abhiroop Sett, Arsalan Hussain, Srestha Tomar, Ashish Kumar Ray, Ranjana Pathania ^#^

^#^ *Address correspondence to* Ranjana Pathania: ranjana.pathania@bt.iitr.ac.in

**S1. Genomic complementation of *hfq* variants**

Genomic complementation was carried out using a modified homologous recombination-based method as previously described (1). In brief, for genomic complementation of WT Hfq and F39A 150bp internal primers corresponding to the US and DS 150bp regions were used to amplify the genomic constructs to be used for integrating the Hfq into the genome of *A. baumannii* Δ*hfq* strain. The chimeric PCR product was concentrated to 5µg in 5µL and transformed into *A. baumannii* ATCC17978 VU Δ*hfq* electrocompetent cells with pAT02 (induced by 5mM isopropyl-β-D-thiogalactopyranoside [IPTG]). The transformants were screened for positive knock-in using the primers Hfq CDS FP and Hfq CDS RP, keeping genomic DNA from Δ*hfq* strain as control. The apramycin cassette was removed from the positive transformants by using pAT03 expressing FLP recombinase. The variants were confirmed using PCR and the amplicons were resolved on a 1% agarose gel and were subsequently sequenced.

**S2. CCCP-exposed persistence assay**

This assay was carried out as per previously reported protocols with some modifications (5). In brief, overnight cultures of WT *A. baumannii* ATCC17978 were sub-cultured in four different culture tubes and incubated at 37°C with shaking till they reached OD600 = 0.5. The cells from two of the tubes were then exposed 0.5X MIC Carbonyl cyanide m-chlorophenyl hydrazone (CCCP) at a final concentration of 64 mg/L. Both the exposed and unexposed tubes were again incubated at 37°C with shaking for 3h. Post incubation the cells were harvested by centrifugation at 5000 RPM for 3 mins and washed twice in 1X sterile PBS. The cells were pelleted and resuspended in sterile LB broth, and their OD 600 was adjusted to 0.5. One tube from each of the CCCP-exposed and unexposed cultures was treated with 50X MIC of cefepime and a time-kill assay was performed by enumerating CFU counts at different time points till 24h for all the cultures, i.e., cefepime treated and untreated.

**S3. Persister inheritability** **assay**

To check for inheritability of persister phenotype, persister cells surviving the antibiotic treatment up to 24 h were harvested, washed with PBS and regrown for 24 h at 37 °C in LB broth without cefepime. The culture was diluted 1:100 with fresh LB broth to obtain mid-log phase cells (O.D. 0.5) and again treated with cefepime up to 24 h. This cycle was repeated three times and surviving antibiotic-treated cells from each passage were enumerated at 24h and also at the 0h time point. To determine the percentage of surviving persister cells, the CFU count post-treatment at 24h was divided by the CFU count at 0h and multiplied by 100. The percentage survival for both WT and the Δ*hfq* cells remained similar throughout the three passages.

**S4. Cellular energetics assays**

**S4.1. Assay for determining intracellular ATP**

BacTiter-Glo kit (Promega) was used to determine the intracellular ATP levels as per the manufacturer’s protocol. In brief, the WT and mutant strains of *A. baumannii* were sub-cultured and allowed to grow till OD_600_ of 0.6 at 37 ⁰C with shaking (180 RPM) in LB broth. 1 mL aliquots of the cells were washed twice in sterile 1X PBS, and one of the WT cell aliquot was subjected to 5µM CCCP treatment for 30 minutes. The cells were then added to 96-well round bottom white well plate (Corning) and to it the BacTiter-Glo reagent was added and mixed. The luminescence was measured using a multimode plate reader (Synergy H1). The luminescence was normalized by first subtracting ATP levels in the spent media and then normalizing the values by dividing by the absorbance read at 600 nm. The normalized values for each of the strains were plotted as values relative to the WT.

**S4.2. Assay for measurement of membrane potential**

Exponential phase *A. baumannii* ATCC17978 cells and genome-complemented Hfq mutants grown in LB were washed and adjusted to an OD600 of ~0.3 in 1X PBS. A 1 mL aliquot of the WT cells was treated with 20µM CCCP (Sigma) as a positive control (data not shown), which was washed again after 30 mins and cells were resuspended in 1X PBS. Next, 1 mL aliquots of each of these cells were incubated with DiBAC4 at 10 μM for 30 minutes, followed by three washing steps with 1X PBS. The cells were then added to the wells of a 96-well opaque plate (Thermo) and fluorescence was recorded at an excitation/emission wavelength of 490/516 nm using a multimode plate reader (Synergy H1). RFU was calculated by normalizing against OD_600_, and the relative change in fluorescence in each of the strains compared to the WT was plotted.

**S4.3. Assessment of cellular metabolic status via estimation of cellular NADH levels**

**S4.3.1. MTT assay**

Exponential phase *A. baumannii* ATCC17978 cells and genome-complemented Hfq mutants grown in LB were washed and adjusted to an OD600 of ~0.3 in 1X PBS. A 1 mL aliquot of the WT cells was treated with 25% DMSO (Himedia) as a positive control (data not shown), which was washed again after 30 mins and cells were resuspended in 1X PBS. Next, 1 mL aliquots of each of these cells were incubated with DiBAC4 at 10 μM for 30 minutes, followed by three washing steps with 1X PBS. The cells were then harvested and resuspended in 100 µL of 1X PBS containing 0.5 mg/mL MTT (Biobasic) dye. The MTT-cell suspension was then added to each well and incubated further for 4h. Following incubation, MTT containing medium was removed and 100 µL of DMSO was added to dissolve the formazan crystals by gentle agitation. The OD was measured at 570 nm in a plate reader (Biotek). Absorbance values were normalized against the OD600 values and plotted with respect to the WT reads.

**S4.3.2. Resazurin assay**

Exponential phase *A. baumannii* ATCC17978 cells and genome-complemented Hfq mutants grown in LB were washed and adjusted to an OD600 of ~0.3 in 1X PBS. These 0.3 OD600 cells were harvested and resuspended in 1mL PBS containing 2 μM resazurin (Sigma), which is a respiration indicator dye. The cell suspension was incubated at 37 °C for 2 h. NADH/H^+^ levels were estimated in terms of fluorescence of the reduced product of resazurin, measured at 530 nm excitation and 590 nm emission. Fluorescence values were normalized to the cells’ optical density at 600 nm. All the values for each strain were plotted with respect to the WT reads.

**S5. Estimation of cell survival from phagocytosis**

All experiments using human blood-derived neutrophils under protocol IITR/IIC/22/2-09 were reviewed and approved by the Institute Human Ethics Committee (HEC) of the Indian Institute of Technology Roorkee. Blood samples were collected from healthy human male and female volunteers, as per the approved guidelines at the Institute hospital. Neutrophils were isolated from human blood using Polymorphprep (ProteoGenix) according to the manufacturer’s instruction and diluted to obtain a final concentration of 1x10 ^4 cells/well. A freshly streaked colony of WT T6- and WT T6+ strain on LB-agar plates were used to inoculate in 5 mL of LB-medium for O/N at 37 ⁰C with shaking. 0.1% inoculum from the O/N cultures was then subcultured into fresh 5 mL of LB-medium and grown at 37 ⁰C with shaking to an OD 600 of 0.6 (mid-log phase). Bacterial cells were harvested and diluted to obtain 10^4 CFU/µL. The diluted bacterial cultures were then opsonized in fetal bovine serum (non-heat treated) for 15 min. Neutrophils were then co-incubated with bacterial strains at an MOI of 1:1 ratio in RPMI 1640 cell culture medium (HIMEDIA) and incubated at 37 ⁰C in an animal tissue culture incubator (Eppendorf). An untreated control was kept where mid-log phase opsonized cells were incubated at 37 ⁰C in RPMI 1640 cell culture medium (HIMEDIA). Post-incubation, samples were serially diluted plated onto LB agar plates and bacterial CFU count was enumerated. The relative percentage survival was calculated by dividing the CFU count post-treatment by the untreated control and multiplying the result with 100.

**S6. *A. baumannii* murine pneumonia model to assess virulence**

All animal experiments under protocol BT/IAEC/2017/01 were reviewed and approved by the Institute Animal Ethics Committee of the Indian Institute of Technology Roorkee. Adult (6–8 week old) age-matched female BALB/c mice (n = 6 for each group) were procured. The mice were quarantined for 1 week with free access to food and water along with a 12-h day-night cycle. On the −4 and −1 days of the experiment, the mice were immunocompromised *via* intraperitoneal injection of cyclophosphamide (150 mg/Kg body wt.) (TCI). On day 0, the mice were anaesthetized with a 1:1 dose of ketamine (75 mg/Kg body wt; IP) (Themis Medicare) and xylazine (16 mg/Kg body wt; IP) (Indian Immunologicals). Subsequently, the mice were infected intranasally with *A. baumannii*, and CFU was enumerated as previously described. Briefly, the mice were anaesthetized and infected intranasally with 20 μl of inoculum containing 2 × 10ˆ9 CFU of the indicated strains. The mice were infected with each of the three strains and divided into two sub-groups (n=6). After 36h of infection, one of the sub-groups for each strain was treated with two doses of cefepime (100 mg/Kg) subcutaneously at an 8h interval. The untreated group was subjected to mock saline subcutaneous injections at similar intervals. After 8h of the second dose, the animals were euthanized, and the lungs were harvested, immediately transferred on ice, and washed with ice-cold 1X sterile PBS. The harvested organs were homogenized to enumerate the bacterial burden by serially diluting and plating on Leeds *Acinetobacter* agar plates (Himedia). The minimum inhibitory concentrations of these strains against cefepime were determined post-enumeration. To perform tissue histology, a single lobe of the lung was removed and imaged after H&E staining by Kaushik Pathology Clinic, Roorkee, India.

**Supporting Tables:**

**Table S1:** List of bacterial strains used in this study.

| **Sl. No.** | **Strain** | **Relevant Characteristics** | **Source/ reference** |
| --- | --- | --- | --- |
| 1 | *Acinetobacter baumannii* ATCC17978 VU | Wild type (WT) *A. baumannii* strain | Purchased from ATCC and VU-type was confirmed in a pervious study (Somok) |
| 2 | *E. coli* DH5α | *supE44 hsdR17 recA1 endA1 gyrA96 thi-1 relA1* | Invitrogen, USA |
| 3. | *E. coli* HST08 | F-, endA1, supE44, thi-1, recA1, relA1, gyrA96, phoA, Φ80d lacZΔ M15, Δ(lacZYA-argF) U169, Δ(mrr-hsdRMS-mcrBC), ΔmcrA, λ- | Takara, Japan |
| 4. | RPT 233 | *Acinetobacter baumannii* ATCC17978 VU *Δhfq* | (2) |
| 5. | RPT393 | *Acinetobacter baumannii* ATCC17978 VU with complemented native Hfq (*c-hfq*) on the genome. | This study |
| 6. | RPT567 | *Acinetobacter baumannii* ATCC17978 with Hfq_F39A_ on the genome. | This study |
| 7. | RPT522 | *E. coli* HST08 carrying the construct for Hfq_F39A_ genomic complementation | This study |
| 8. | RPT392 | *E. coli* DH5α carrying the construct for native Hfq (*c-hfq*) genomic complementation | This study |

**Table S2.** List of plasmids used in this study

| Sl. No. | Plasmid name | Relevant Characteristics | Source/ reference |
| --- | --- | --- | --- |
| 1 | pAT02 | Plasmid expressing *A. baumannii* RecT homolog. *amp^r^* | (1) |
| 2 | pAT03 | Plasmid expressing FLP recombinase enzyme (flippase) for expression in *A. baumannii*. *amp^r^* | (1) |
| 3. | pMDIAI | Plasmid containing Apramycin resistance cassette between FRT sites | Addgene, USA |
| 4. | pUC18 | pUC18 vector for cloning *hfq* knock-in constructs. *amp^r^* | Addgene, USA |
| 5. | pUC-WT-KI | pUC18 harbouring 5’-upstream (US) 500 bp region of *hfq*, WT *hfq* CDS, Apramycin-FRT cassette, and 3’ downstream (DS) 500bp region of *A. baumannii hfq. apr^r^* | (3) |
| 6. | pUC-F39A-KI | pUC18 harbouring 5’-upstream (US) 500 bp region of *hfq*, *hfq_F39A_* CDS, Apramycin-FRT cassette, and 3’ downstream (DS) 500bp region of *A. baumannii hfq. apr^r^* | This study |

**Table S3:** List of Oligos used in this study.

| **Sl. No.** | **Primer name** | **Sequence (5**' **- 3**'**)** | **Description** |
| --- | --- | --- | --- |
| 1 | RT *tssB* FP | TCAGCGAATTCGACCTCCAC | qRT-PCR forward primer for *tssB* |
| 2 | RT *tssB* RP | GTACGCTCAAGCTCAGATGC | qRT-PCR reverse primer for *tssB* |
| 3. | RT *tssC* FP | GTTGGTGTGCTGCTATTCGC | qRT-PCR forward primer for *tssC* |
| 4. | RT *tssC* RP | CTCTTTTTCACGGCGATCCG | qRT-PCR reverse primer for *tssC* |
| 5. | RT *hcp* FP | CTTCAAGTAGTGTAGGCGGC | qRT-PCR forward primer for *hcp* |
| 6. | RT *hcp* RP | CCATTTGCACGATAGAAGTC | qRT-PCR reverse primer for *hcp* |
| 7. | RT *tssE* FP | GTGGGGCTTTCTACAGCCAA | qRT-PCR forward primer for *tssE* |
| 8. | RT *tssE* RP | ACCCGTATTTGTCTTAGCCGAG | qRT-PCR reverse primer for *tssE* |
| 9. | RT *tssF* FP | TAGTAGCTTGGCGAGACGTG | qRT-PCR forward primer for *tssF* |
| 10. | RT *tssF* RP | GATCACACGCCACTGTTCAC | qRT-PCR reverse primer for *tssF* |
| 11. | RT *tssM* FP | CTCCGGCAACCAATCAGTCT | qRT-PCR forward primer for *tssM* |
| 12. | RT *tssM* RP | AGCTGTAATACGAGCACCCG | qRT-PCR reverse primer for *tssM* |
| 13. | RT *tssH* FP | CTCGAGTGCAATTATGCAGGC | qRT-PCR forward primer for *tssH* |
| 14. | RT *tssH* RP | CACAACTCTCATGCGCCCTA | qRT-PCR reverse primer for *tssH* |
| 15. | T2SS gspI RTF | ACGCAGTCCACTTCAAATGC | qRT-PCR forward primer for *gspI* |
| 16. | T2SS gspI RTR | CCTGCTTACTTTGAGTTCCTTGC | qRT-PCR reverse primer for *gspI* |
| 17. | T2SS gspJ RTF | TCACAAAATGGCCCGAGGTT | qRT-PCR forward primer for *gspJ* |
| 18. | T2SS gspJ RTR | ACCAGCGACTGTAAGTTGGA | qRT-PCR reverse primer for *gspJ* |
| 19. | AB-NuoC RTF | CGTATCCGTACACCGACGTT | qRT-PCR forward primer for *nuoC* |
| 20. | AB-NuoC RTR | TCCACGTCAGCCATAACCAC | qRT-PCR reverse primer for *nuoC* |
| 21. | AB-NuoG RTF | CTTGTACCATTTGCGTGGGC | qRT-PCR forward primer for *nuoG* |
| 22. | AB-NuoG RTR | CGGTCGAATAAACGAACGCC | qRT-PCR reverse primer for *nuoG* |
| 23. | AB-NuoE RTF | GCTTGCTTCTTGATGGGTGC | qRT-PCR forward primer for *nuoE* |
| 24. | AB-NuoE RTR | CAGCAAATTGGAAGCAGCGT | qRT-PCR reverse primer for *nuoE* |
| 25. | AB-NuoF RTF | ATGGTGGTAGACGAGACCCA | qRT-PCR forward primer for *nuoF* |
| 26. | AB-NuoF RTR | CAAACCATCACGGCAAGGTG | qRT-PCR reverse primer for *nuoF* |
| 27. | cytochrome ubiq oxi I RTF | TAAGTACGGGCGATGTGTGG | qRT-PCR forward primer for *Cytochrome o ubiquinol*  *oxidase (subunit I)* |
| 28. | cytochrome ubiq oxi I RTR | GTGAGCTTGGACCTAAGCGA | qRT-PCR reverse primer for *Cytochrome o ubiquinol*  *oxidase (subunit I)* |
| 29. | cytochrome o ubiq oxi III RTF | CTTTGGTAGGTACGCACGGT | qRT-PCR forward primer for *Cytochrome o*  *ubiquinol oxidase (subunit III)* |
| 30. | cytochrome o ubiq oxi III RTR | AGCAAGCAAGACGACGTGTA | qRT-PCR reverse primer for *Cytochrome o*  *ubiquinol oxidase (subunit III)* |
| 31. | ubiq oxi II RTF | ACCAAGCTGAATTGGCAACC | qRT-PCR forward primer for *Ubiquinol II Oxidase* |
| 32. | ubiq oxi II RTR | GCAAGCGCTTCTTGATCACC | qRT-PCR reverse primer for *Ubiquinol II Oxidase* |
| 33. | Malate synt RTF | GGCTTAATGGAAGACCGTGC | qRT-PCR forward primer for *Malate Synthase* |
| 34. | Malate synt RTR | TTGCCATGCGTTTAAGCACT | qRT-PCR reverse primer for *Malate Synthase* |
| 35. | isocitrate lyase RTF | ACCACACTGCTGCTCTTTCT | qRT-PCR forward primer for *Isocitrate lyase* |
| 36. | isocitrate lyase RTR | CGATACCGCCACGGATTTCT | qRT-PCR reverse primer for *Isocitrate lyase* |
| 37. | Fumarase RTF | CATTAGGTGGCACAGCGGTA | qRT-PCR forward primer for *Fumarase* |
| 38. | Fumarase RTR | AGGAGCGGTAACAAATGGCA | qRT-PCR reverse primer for *Fumarase* |
| 39. | Isocitrate dehy RTF | ATGGGGTGGATAGAAGCAGC | qRT-PCR forward primer for *Isocitrate dehydrogenase* |
| 40. | Isocitrate dehy RTR | GCAACGTAACAAGGTCGCTC | qRT-PCR reverse primer for *Isocitrate dehydrogenase* |
| 41. | Pyruvate dehy E1 RTF | GCCTACACCCACTTGCTGAA | qRT-PCR forward primer for *Pyruvate dehyrogenase* |
| 42. | Pyruvate dehy E1 RTR | TGGTCGGTCGCAGAAACTAC | qRT-PCR reverse primer for *Pyruvate dehyrogenase* |
| 43. | Acetyl ornithine transaminase RTF | GCAATTCGTGGCAGTGGTTT | qRT-PCR forward primer for *Acetyl ornithine transferase* |
| 44. | Acetyl ornithine transaminase RTR | CCTTCTTCACCCGCAAGGTT | qRT-PCR reverse primer for *Acetyl ornithine transferase* |
| 45. | branched aa transaminase RTF | TCCAGATATTGCGGGTGGTG | qRT-PCR forward primer for *Branched chain aminotransferase* |
| 46. | branched aa transaminase RTR | CGCGTAATACGACGCTCAAC | qRT-PCR reverse primer for *Branched chain aminotransferase* |
| 47. | AprF-Bam | ATCAGGATCCGTCGACCTGCAGTTC | Apramycin gene amplification for cloning knock out construct in pUC 18 plasmid |
| 48. | AprR-Kpn | ATGGTACCGTGTAGGCTGGAGCTGCTTC | Apramycin gene amplification for cloning knock out construct in pUC 18 plasmid |
| 49. | FPUS500+hfqCDS_SalI | ATGTCGACATTGCAGGCAGAACAACC | Cloning primers for Upstream 500 bp region of *hfq* along with its CDS (and stop codon) |
| 50. | RPUS500+hfqCDS_BamHI | ATTGGGATCCTTAACGATTGTTTTCGTCG | Cloning primers for Upstream 500 bp region of *hfq* along with its CDS (and stop codon) |
| 51. | FPDS500hfq_Kpn1 | GTGGGTACCCGTTAATTGATTAGCTTGAA | Cloning primers for *hfq* Downstream 500 bp |
| 52. | RPDS500hfq_EcoR1 | GCGGCGAATTCCAAACCTATCCC | Cloning primers for *hfq* Downstream 500 bp |
| 53. | Hfq US 150 FP | CTTACTTGACGATAAAGCAGGC | Primers for PCR construct of Hfq variants to be cloned into the genome |
| 54. | Hfq DS 150 RP | CAGCAAATATTAATACCATTTGCAAAGAA | Primers for PCR construct of Hfq variants to be cloned into the genome |
| 55. | Hfq CDS FP | TCTAAAGGTCAAACTTTACAAG | Primers to screen for successful Hfq knock-in in *A. baumannii Δhfq* genome |
| 56. | Hfq CDS RP | GACTGGTTTTTTTCAAGCTAATC | Primers to screen for successful Hfq knock-in in *A. baumannii Δhfq* genome |
| 57. | Hfq F39A RP | CATATTGGTC*CGC*AGATTCAATATGACCTTGT | Primers to introduce F39A mutation into the WT *hfq* sequence using inverse PCR of pUC-WT-KI and InFusion cloning |
| 58. | Hfq F39A FP | TGAATCT*GCG*GACCAATATGTTGTTTTACTAAAAA | Primers to introduce F39A mutation into the WT *hfq* sequence using inverse PCR of pUC-WT-KI and InFusion cloning |

**Table S4 :** Minimum inhibitory concentration (MIC) of different antibiotics against the wild-type and Δ*hfq* strains of *A. baumannii*. The assay was carried as per CLSI guidelines and the values represented are in mg/L.

| **Table S4: Minimum Inhibitory concentrations of different antibiotics (in mg/L)** | | |
| --- | --- | --- |
| **Antibiotic** | ***A. baumannii* ATCC17978** | ***A. baumannii* ATCC17978 Δ*hfq*** |
| **Rifampicin** | **2** | **1** |
| **Polymyxin B** | **0.125** | **0.125** |
| **Gentamicin** | **4** | **2** |
| **Apramycin** | **4** | **1-0.5** |
| **Amikacin** | **16-32** | **32** |
| **Cefotaxime** | **4** | **2** |
| **Cefepime** | **4** | **2-1** |
| **Ciprofloxacin** | **<0.03125** | **<0.03125** |

**Supporting Figures:**


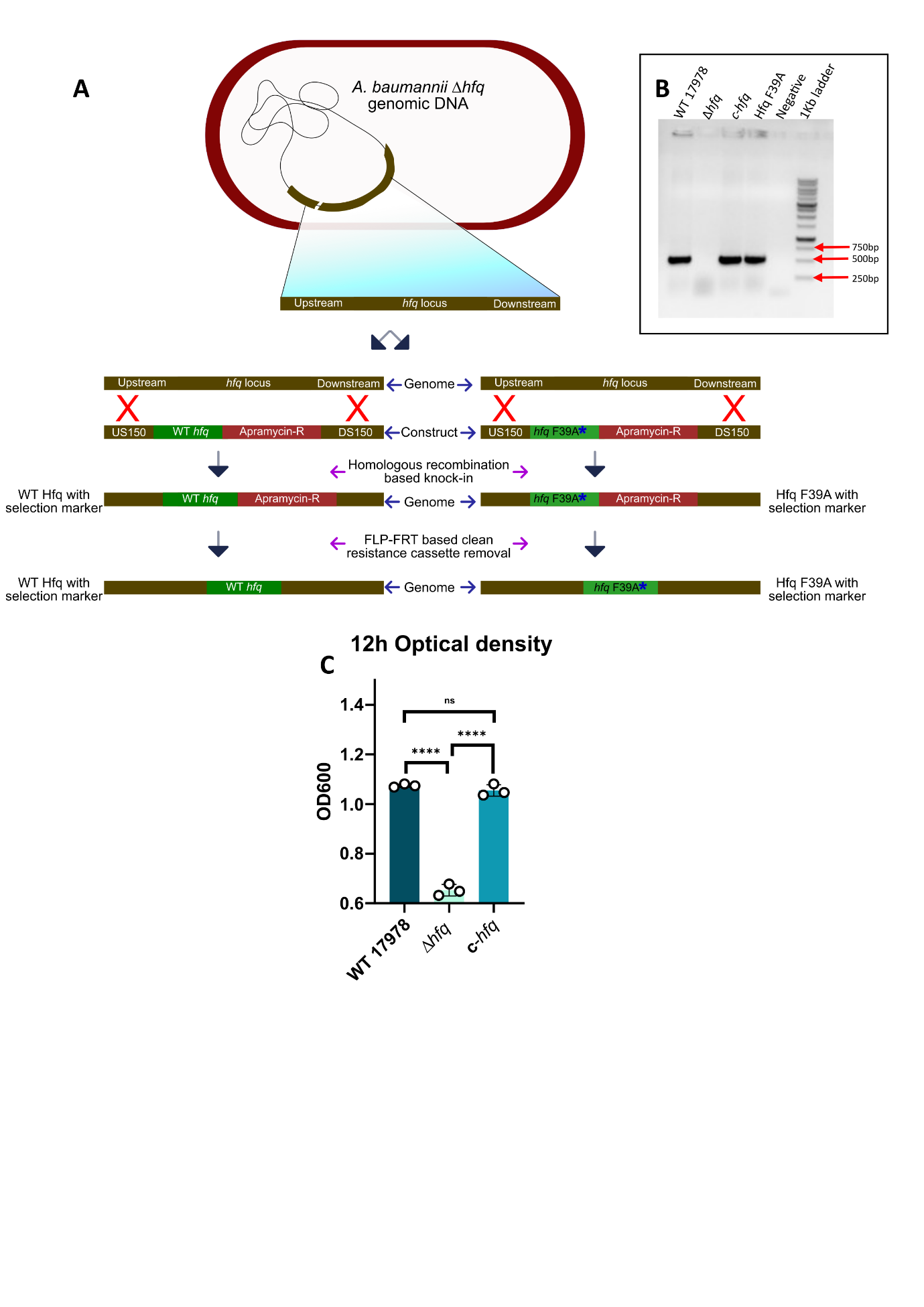


**FIG S1** The WT Hfq and Hfq F39A variants into the genome of *A. baumannii* ATCC17978 VU Δ*hfq* strain. (A) A schematic of the homologous recombination-based approach used to introduce the Hfq WT and F39A coding sequence into the genome. (B) The *A. baumannii* ATCC17978 VU Hfq mutants are confirmed using PCR with primers for the coding region of Hfq and confirmed by Sanger sequencing. The PCR product is resolved on a 1% agarose gel. (C)The optical density of *A. baumannii* WT, Δ*hfq,* and c-*hfq* strains at 12h of growth. Each bar represents the mean of three experiments, and the error bars represent the SD. Statistical significance was determined by one-way ANOVA p-value was ****, p ≤ 0.0001; ns, non-significant. Tukey’s test was used as a post hoc test to determine the statistical significance of all pairs of data.


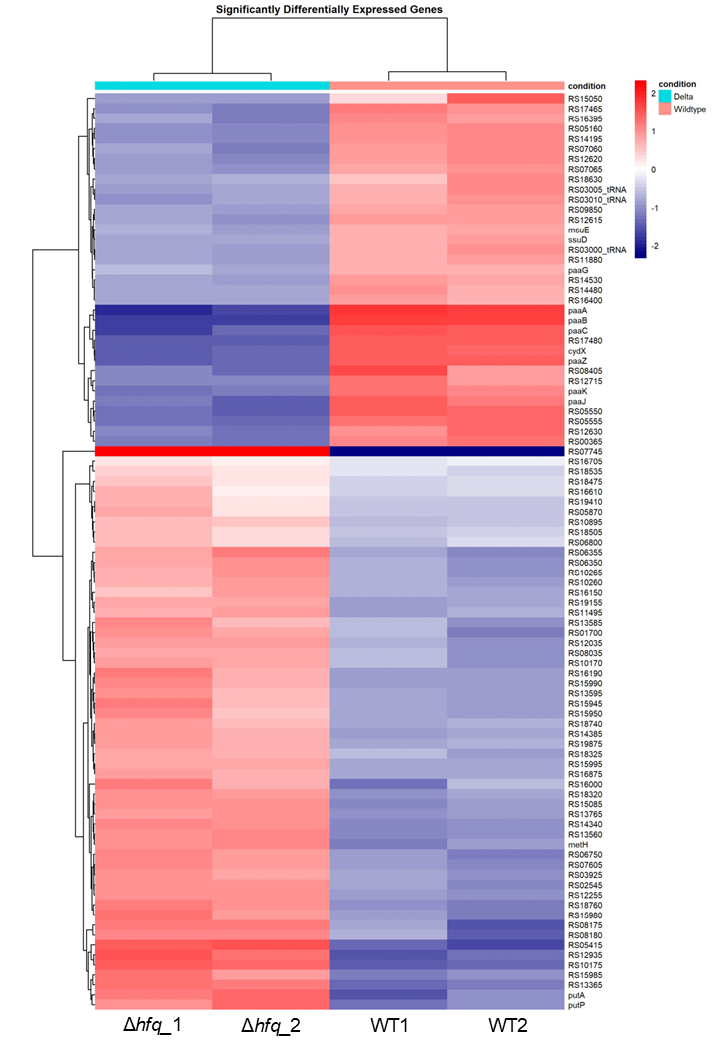


**FIG S2** Deletion of *hfq* leads to differential expression of a number of transcripts. The figure is a representative heat-map the significantly differentially expressed genes between wild-type and the *hfq* deletion mutant (Δ*hfq*) strains with (logFC) > 2. The assay was carried out using biological duplicates.


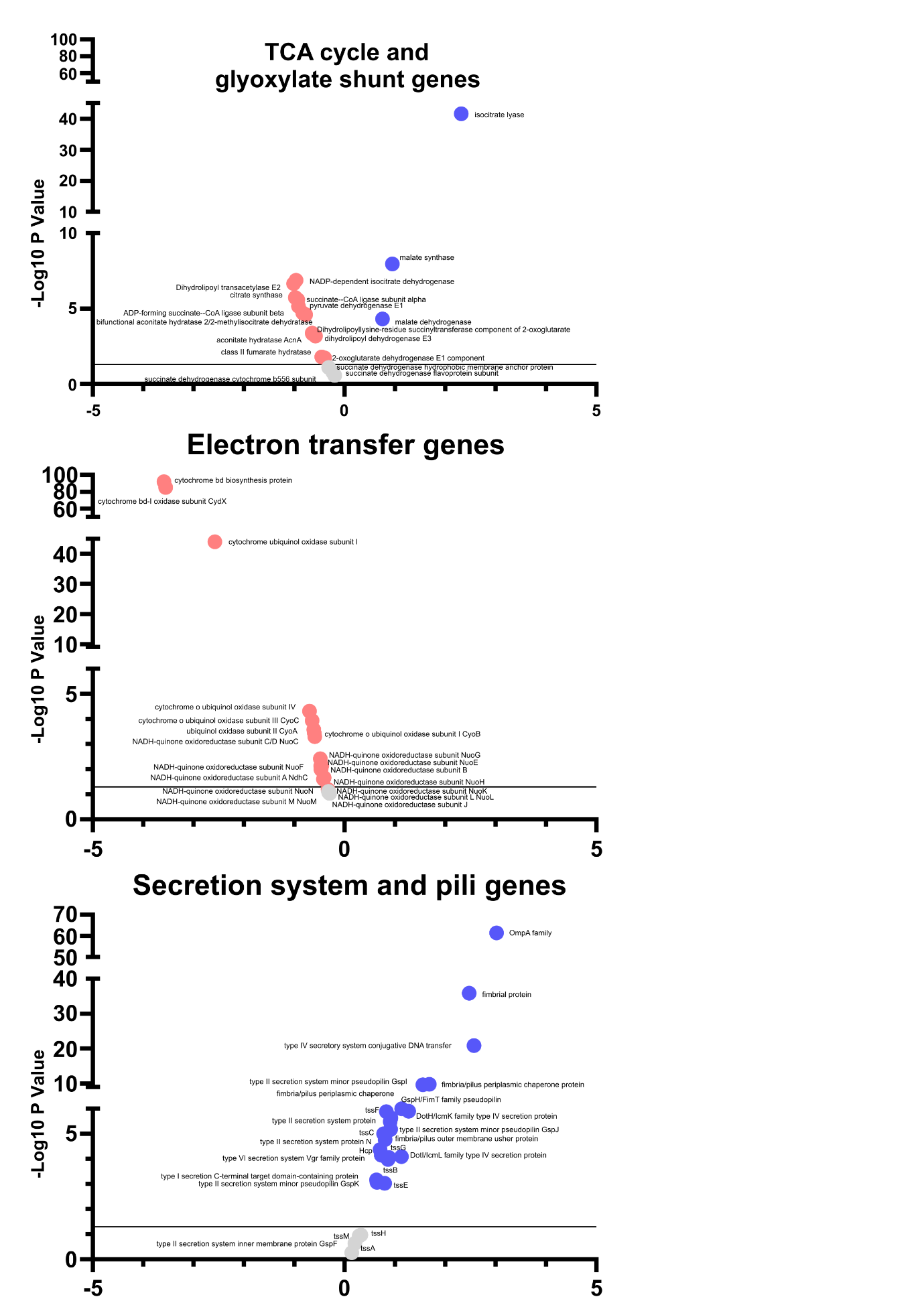


**C**

**B**

**A**

**FIG S3** Deletion of *hfq* results in altered transcriptome. Volcano plots depicting significantly upregulated (pink) and downregulated (blue) transcript levels of different mRNAs. The genes involved in (A) TCA cycle and metabolism, (B) electron transfer and (C) T6SS, T2SS are depicted in these plots. Points depicted in grey below the black are statistically non-significant with a P-value of p >0.05.

**
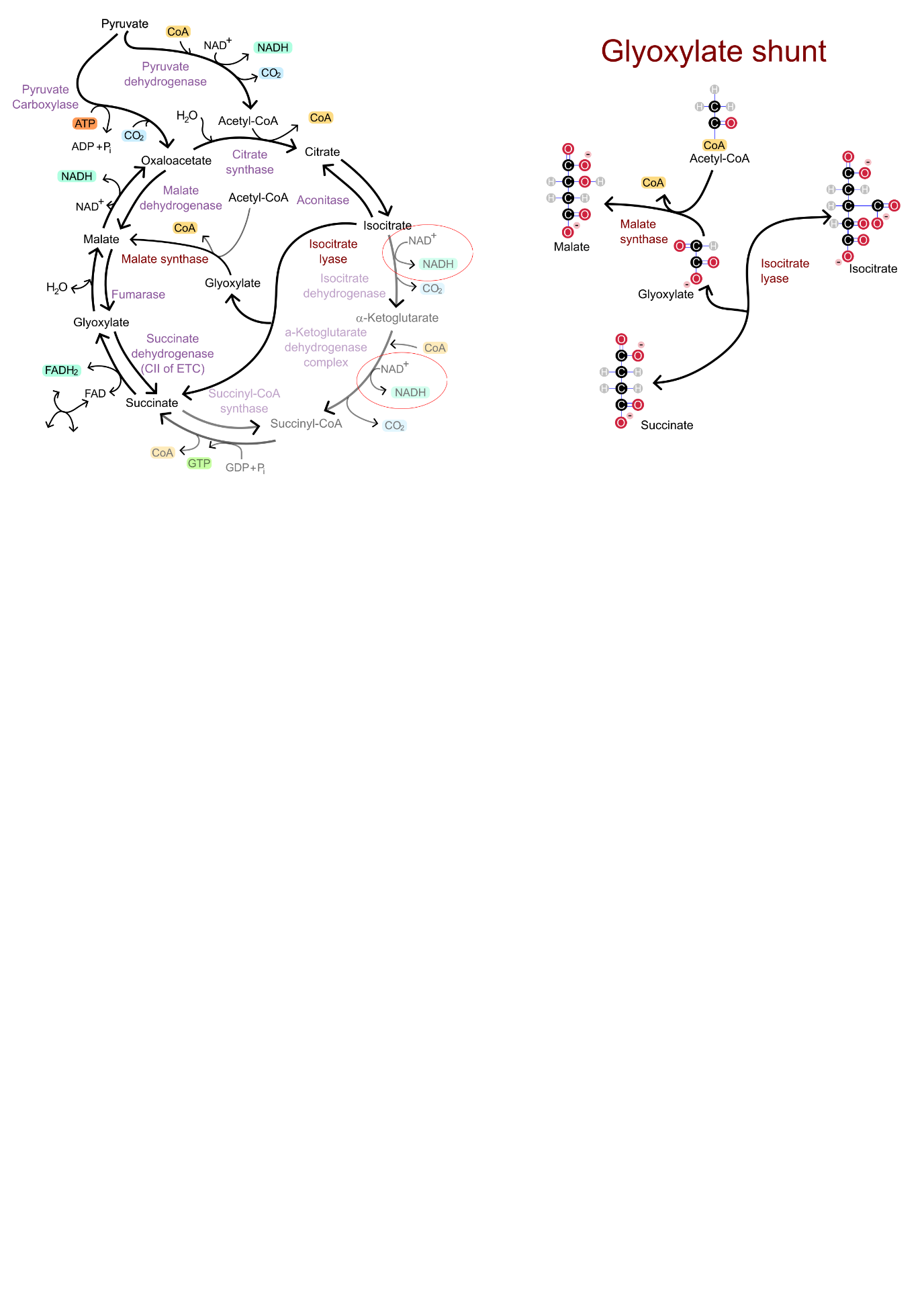
**

**FIG S4** A schematic highlighting the glyoxylate shunt that bypasses multiple NADH-generating steps (marked with red circles) of the TCA cycle.


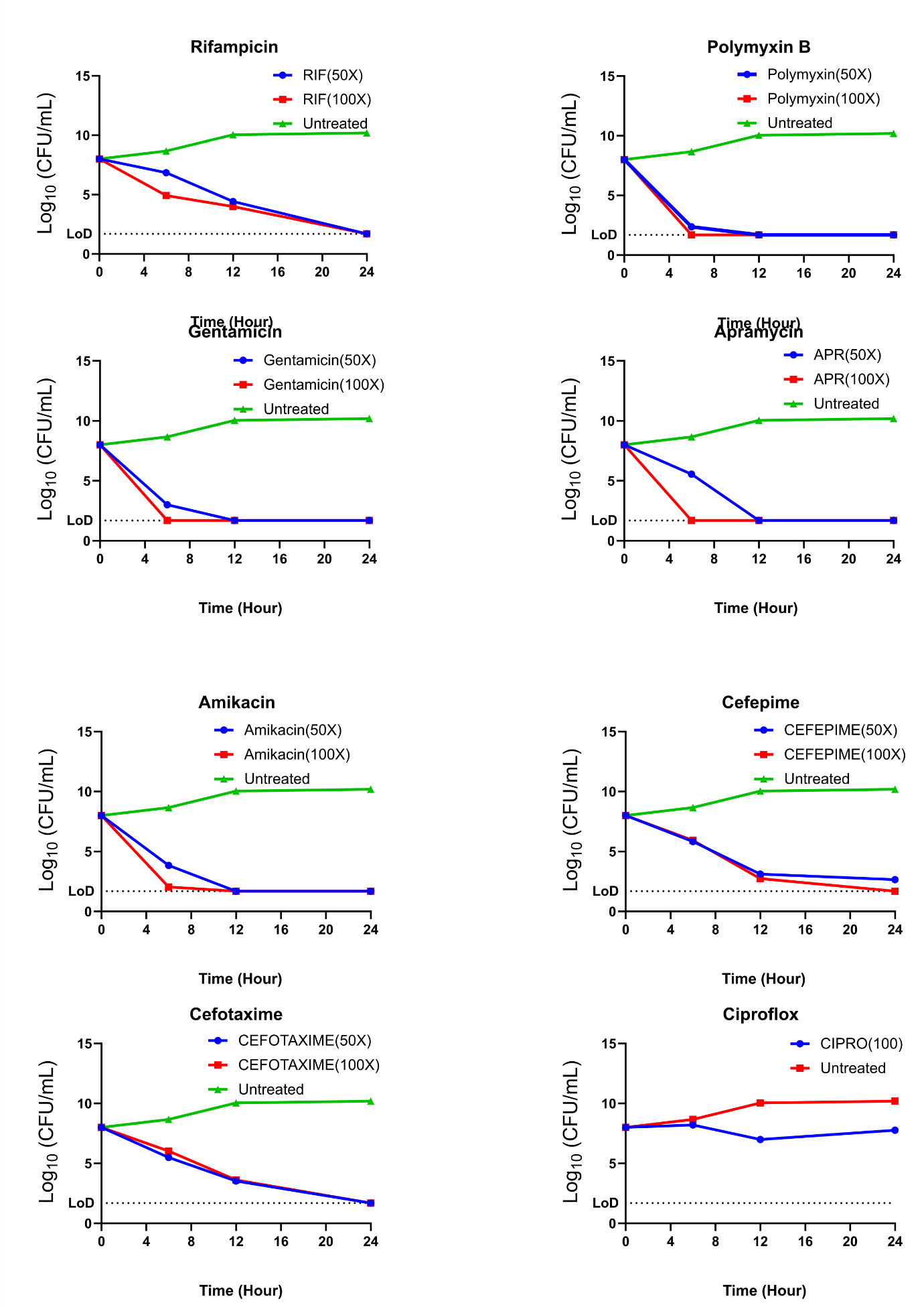


**FIG S5** A screen to identify spontaneous persistence inducing antibiotic concentrations in *A. baumannii*. Bi-phasic kill-curve of *A. baumannii* wild type treated with 50X (blue) and 100X (red) MIC of different antibiotics along with enumeration of CFU counts at different time intervals. The bi-phasic kill-curve is one representative plot of two independent biological replicates.


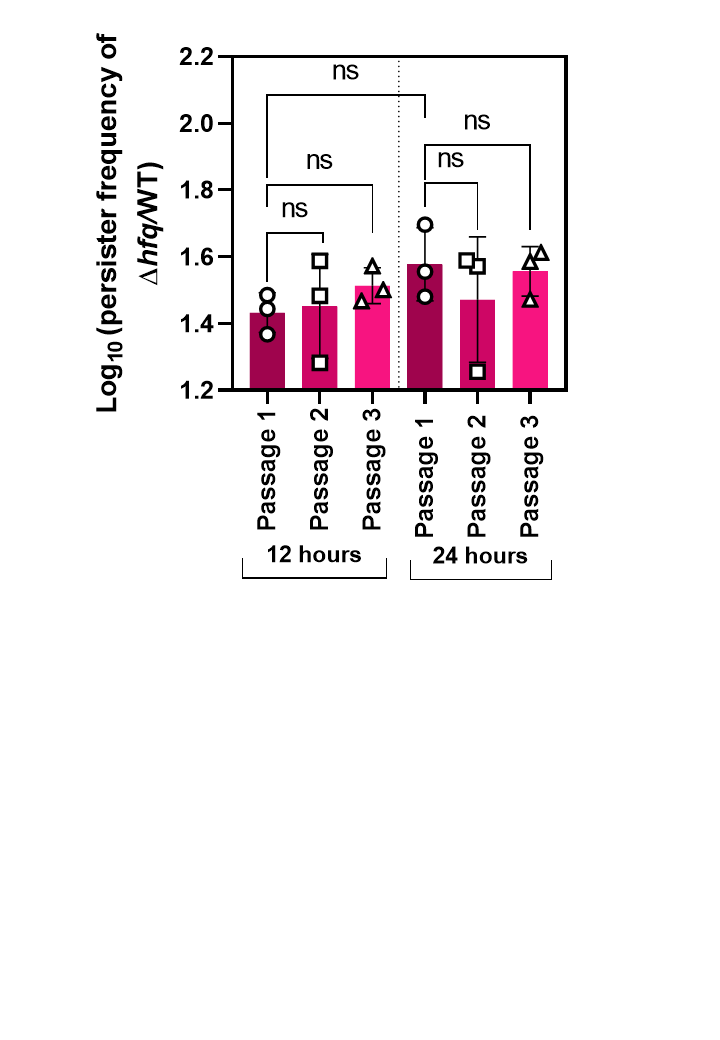


**FIG S6** The cefepime-induced spontaneous persister frequency remains similar even after multiple rounds of treatment. *A. baumannii* mid-log phase WT and Δ*hfq* cells were treated with 50X MIC concentration of cefepime and CFU counts at the end of 12h and 24h was enumerated. Subsequently the surviving persisters were incubated overnight without no treatment followed by sub-culturing and incubating them till the mid-log phase. These cells were again exposed to 50X MIC concentration of cefepime and CFU counts at the end of 12h and 24h was enumerated. This process was carried out thrice and log_10_ (*persister frequency of Δhfq / persister frequency of WT*) was plotted for the three passages. Each bar represents the mean values of three independent biological replicates. Statistical significance was determined by one-way ANOVA p-value was ns, non-significant. Tukey’s test was used as a post hoc test to determine the statistical significance of all pairs of data.


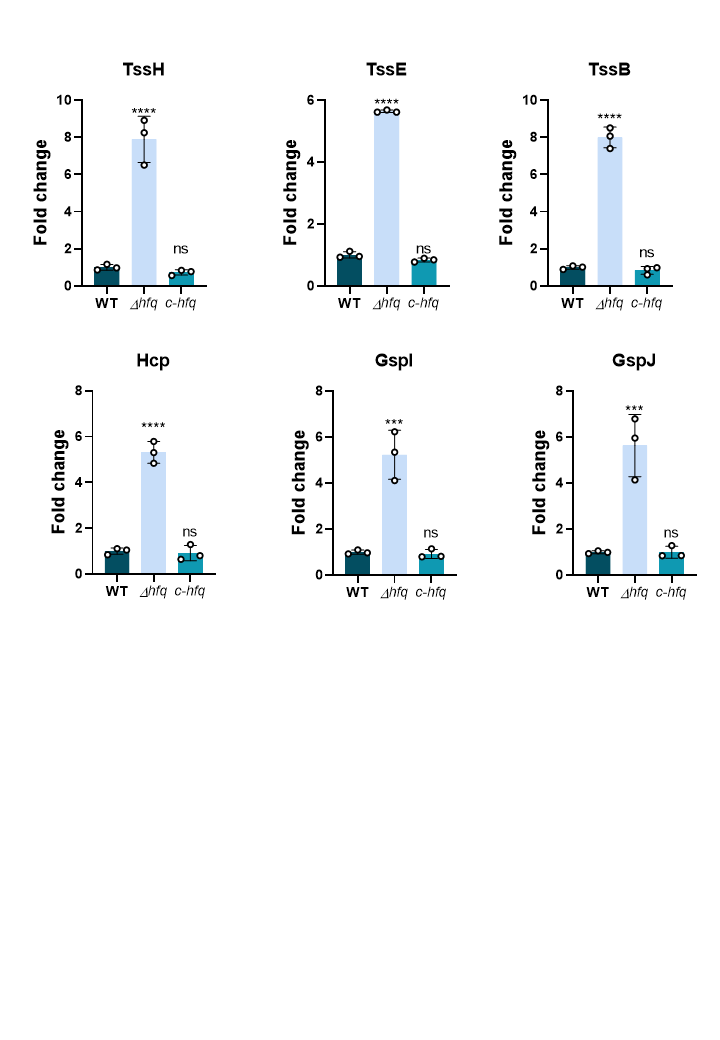


**FIG S7** Various secretion system genes are differentially expressed in cefepime-induced persisters of *A. baumannii* after 24h post treatment. The expression profiles of several Type VI secretion system (T6SS) genes like *tssF, tssC, tssM, tssH, tssE, tssB* and *hcp* along with Type III secretion system (T2SS) genes like *gspI* and *gspJ* was examined by qRT-PCR. Each bar represents the mean values of three independent biological replicates along with SD. Statistical significance was determined by one-way ANOVA p-value was ***, p ≤ 0.001; ****, p ≤ 0.0001; ns, non-significant. Tukey’s test was used as a post hoc test to determine the statistical significance of all pairs of data.


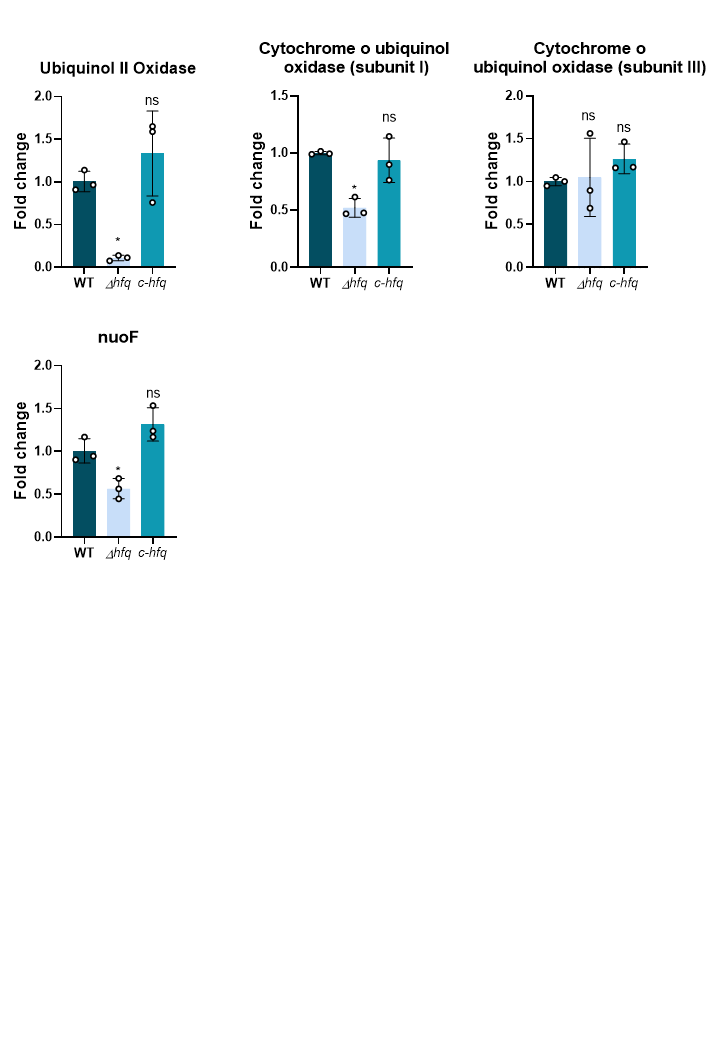


**FIG S8** Various electron-transport genes are differentially expressed in cefepime-induced persisters of *A. baumannii* after 24h post treatment. The expression profiles of several Type VI secretion system (T6SS) genes like Ubiquinol III Oxidase, Cytochrome o ubiquinol oxidase (subunit I), Cytochrome o ubiquinol oxidase (subunit III), and complex I genes (*nuoG, nuoF, nuoC, and nuoE*) was examined by qRT-PCR. Each bar represents the mean values of three independent biological replicates along with SD. Statistical significance was determined by one-way ANOVA p-value was *, p ≤ 0.05; **, p ≤ 0.001; ns, non-significant. Tukey’s test was used as a post hoc test to determine the statistical significance of all pairs of data.


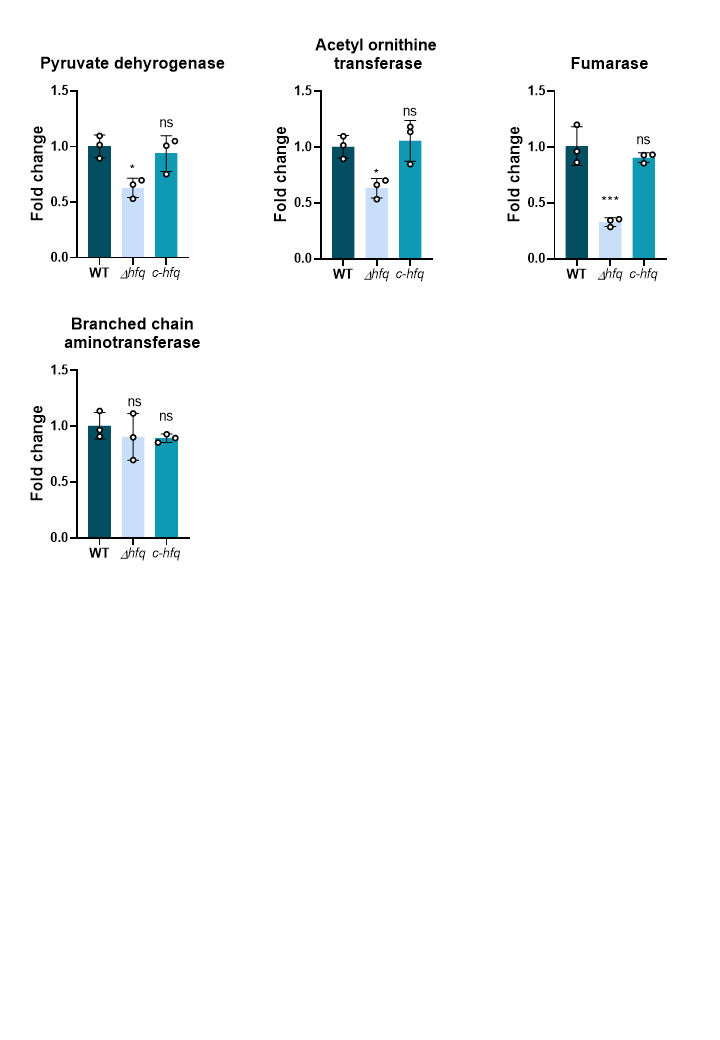


**FIG S9** Various genes of metabolic pathways are differentially expressed in cefepime-induced persisters of *A. baumannii* after 24h post treatment. The expression profiles of several TCA cycle genes (pyruvate dehydrogenase, isocitrate dehydrogenase, and fumerase or *fumC*), genes of glyoxylate shunt (malate synthase, and isocitrate lyase) along with genes of aminotransferases (Acetyl ornithine transferase, and branched chain amino acid aminotransferase) was examined by qRT-PCR. Each bar represents the mean values of three independent biological replicates along with SD. Statistical significance was determined by one-way ANOVA p-value was *, p ≤ 0.05; **, p ≤ 0.001; ***, p ≤ 0.001; ****, p ≤ 0.0001; ns, non-significant. Tukey’s test was used as a post hoc test to determine the statistical significance of all pairs of data.


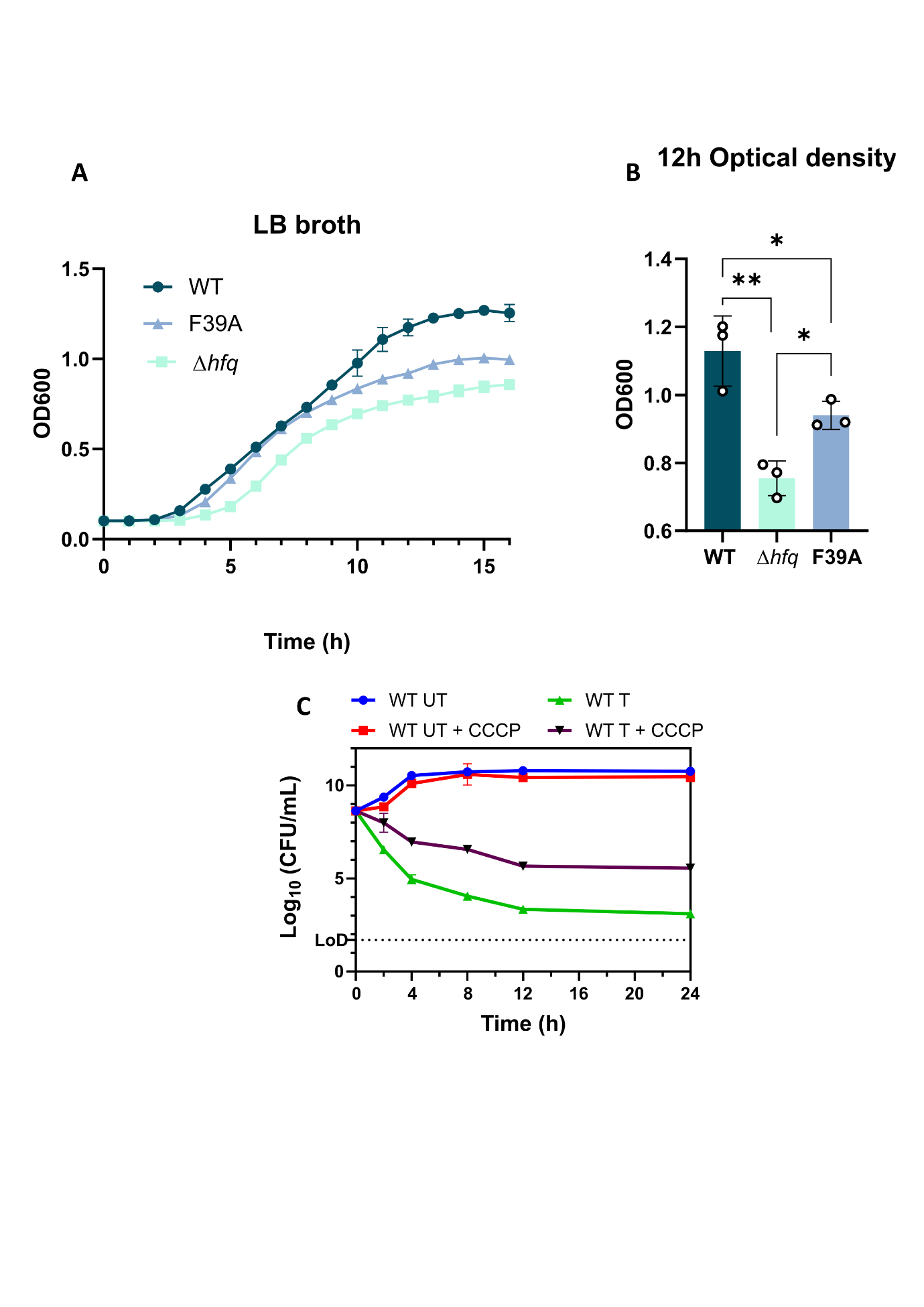


**FIG S10** Impact of RNA-binding face mutations of Hfq (A) A growth profile of *A. baumannii* WT, Δ*hfq,* and Hfq_F39A_ strains grown in LB broth at 37⁰C at 0.5-hour intervals. Each point represents the mean of four values with SD shown as error bars. The growth phenotype assay is a representative plot of three independent biological replicates. (B)The opticall density of *A. baumannii* WT, Δ*hfq,* and Hfq_F39A_ strains at 12h of growth. (C) Bi-phasic kill-curve of *A. baumannii* wild-type cells pre-exposed with CCCP (0.5X MIC) vs without pre-exposure treated with 50X MIC of cefepime along with enumeration of CFU counts at different time intervals. The bi-phasic kill-curve is one representative plot of two independent biological replicates. Each bar represents the mean values of three independent biological replicates along with SD. Statistical significance was determined by one-way ANOVA p-value was *, p ≤ 0.05; **, p ≤ 0.001. Tukey’s test was used as a post hoc test to determine the statistical significance of all pairs of data.


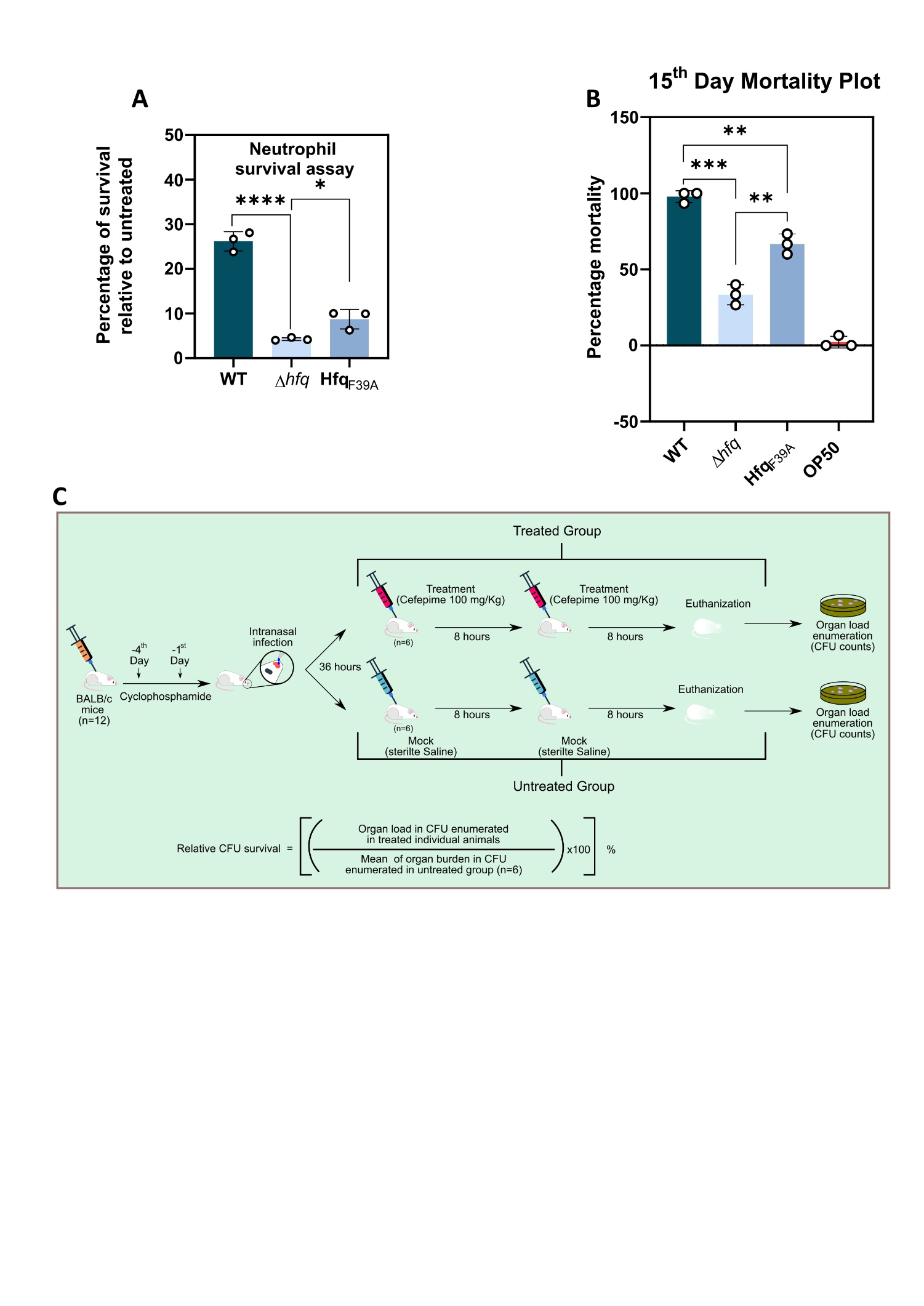


**FIG S11** Impact of RNA-binding face mutations of Hfq (A) Mid-log phase bacterial strains were co-incubated with neutrophils for 4 h at an MOI of 1:1. The percentage of bacterial survival was enumerated, accounting for the respective untreated control (without neutrophils) as 100%. (B) 15^th^-day mortality percentage of *C. elegans* (strain AU37) feeding on *E. coli* OP50 was taken as a negative control and was excluded from the statistical analysis. The percentage survival of worms was determined by counting the number of live worms in each plate at the end of 15^th^-day of feeding. (C) A schematic overview of the experimental work-flow to evaluate relative clearance efficiency of each of the *A. baumannii* strains used in this study. Each bar represents the mean of three experiments, and the error bars represent the SD. Statistical significance was determined by one-way ANOVA p-value was*, p ≤ 0.05; **, p ≤ 0.001; ***, p ≤ 0.001; ****, p ≤ 0.0001. Tukey’s test was used as a post hoc test to determine the statistical significance of all pairs of data.

**References:**

1. Tucker AT, Nowicki EM, Boll JM, Knauf GA, Burdis NC, Trent MS, Davies BW. Defining gene-phenotype relationships in Acinetobacter baumannii through one-step chromosomal gene inactivation. mBio. 2014 Aug 5;5(4):e01313-14. doi: 10.1128/mBio.01313-14.

2. Sharma A, Dubey V, Sharma R, Devnath K, Gupta VK, Akhter J, Bhando T, Verma A, Ambatipudi K, Sarkar M, Pathania R. The unusual glycine-rich C terminus of the Acinetobacter baumannii RNA chaperone Hfq plays an important role in bacterial physiology. J Biol Chem. 2018 Aug 31;293(35):13377-13388. doi: 10.1074/jbc.RA118.002921.

3. Sett A, Maiti PK, Garg K, Hussain A, Saini S, Pandey S, Pathania R. 'GGFGGQ' repeats in Hfq of Acinetobacter baumannii are essential for nutrient utilization and virulence. J Biol Chem. 2024 Dec;300(12):107895. doi: 10.1016/j.jbc.2024.107895.

4. Zhang A, Schu DJ, Tjaden BC, Storz G, Gottesman S. Mutations in interaction surfaces differentially impact E. coli Hfq association with small RNAs and their mRNA targets. J Mol Biol. 2013 Oct 9;425(19):3678-97. doi: 10.1016/j.jmb.2013.01.006. Epub 2013 Jan 11.

5. Grassi L, Di Luca M, Maisetta G, Rinaldi AC, Esin S, Trampuz A, Batoni G. Generation of Persister Cells of Pseudomonas aeruginosa and Staphylococcus aureus by Chemical Treatment and Evaluation of Their Susceptibility to Membrane-Targeting Agents. Front Microbiol. 2017 Oct 4;8:1917. doi: 10.3389/fmicb.2017.01917.
